# Tracking immunodynamics by identification of S-G_2_/M-phase T cells in human peripheral blood^☆^^☆☆^

**DOI:** 10.1016/j.jaut.2020.102466

**Published:** 2020-08

**Authors:** Miguel Muñoz-Ruiz, Irma Pujol-Autonell, Hefin Rhys, Heather M. Long, Maria Greco, Mark Peakman, Tim Tree, Adrian C. Hayday, Francesca Di Rosa

**Affiliations:** a Immunosurveillance Laboratory, The Francis Crick Institute, London, UK; b Peter Gorer Department of Immunobiology, King's College London, London, UK; c National Institute for Health Research (NIHR) Biomedical Research Center (BRC), Guy's and St Thomas' NHS Foundation Trust and King's College London, London, UK; d Flow Cytometry Science Technology Platform, The Francis Crick Institute, London, UK; e Institute of Immunology and Immunotherapy, University of Birmingham, Birmingham, UK; f Genomics Equipment Park, The Francis Crick Institute, London, UK; g Institute of Molecular Biology and Pathology, National Research Council of Italy (CNR), Rome, Italy

**Keywords:** Type 1 diabetes, Immuno-monitoring, CD8 T cells

## Abstract

The ready availability of human blood makes it the first choice for immuno-monitoring. However, this has been largely confined to static metrics, particularly resting T cell phenotypes. Conversely, dynamic assessments have mostly relied on cell stimulation *in vitro* which is subject to multiple variables. Here, immunodynamic insights from the peripheral blood are shown to be obtainable by applying a revised approach to cell-cycle analysis. Specifically, refined flow cytometric protocols were employed, assuring the reliable quantification of T cells in the S-G_2_/M phases of the cell-cycle (collectively termed “T Double S” for T cells in S-phase *in Sanguine*: in short “T_DS_” cells). Without protocol refinement, T_DS_ could be either missed, as most of them layed out of the conventional lymphocyte gates, or confused with cell doublets artefactually displaying high DNA-content. To illustrate the nature of T_DS_ cells, and their relationship to different immunodynamic scenarios, we examined them in healthy donors (HD); infectious mononucleosis (IM) patients versus asymptomatic EBV^+^ carriers; and recently-diagnosed T1D patients. T_DS_ were reproducibly more abundant among CD8^+^ T cells and a defined subset of T-regulatory CD4^+^ T cells, and were substantially increased in IM and a subset of T1D patients. Of note, islet antigen-reactive T_DS_ cell frequencies were associated with an aggressive T cell effector phenotype, suggesting that peripheral blood can reflect immune events within tissues in T1D, and possibly in other organ-specific autoimmune diseases.

Our results suggest that tracking T_DS_ cells may provide a widely applicable means of gaining insight into ongoing immune response dynamics in a variety of settings, including tissue immunopathologies where the peripheral blood has often not been considered insightful.

## Highlights

• “T_DS_” cells (i.e. in S-G_2_/M phases of cell cycle) are present in peripheral blood.
• T_DS_ cells are represented to different degrees in different T cell subtypes.
• T_DS_ cells were associated with activated effector functions in T1D patients.
• T_DS_ analyses can potentially functionally refine immuno-monitoring in many settings.
• Abstract.

## 1 Introduction

Clonal expansions of adaptive lymphocytes underpin host-beneficial responses to infection, vaccination, and cancer, yet can be responsible for graft rejection and for self-antigen targeting in chronic autoimmune diseases, such as Type 1 Diabetes (T1D). In all these scenarios, it would be useful to assess the status and dynamics of ongoing adaptive immune responses, for which peripheral blood is the most utilitarian source [1]. Indeed, the potential of peripheral blood to reflect responses has been highlighted by growing imperatives to monitor cancer immunotherapy [2]. Thus, peripheral blood immuno-monitoring has been increasingly applied, in parallel with quantum increases in analytical capabilities such as single cell transcriptomics and multiparameter mass cytometry [[3], [4], [5]].

Given that clonal behavior underpins adaptive immunity, single cell resolution is essential for monitoring immune responses. Although this is a power of flow cytometry, peripheral blood analyses have been limited almost exclusively to “snap-shot” assessments of cell phenotypes, with the cells’ dynamic status only revealed by re-stimulation *in vitro*, which is widely acknowledged to be influenced by many variables. We therefore revisited this issue, choosing to re-evaluate peripheral blood cell-cycle status.

Peripheral blood T cells have been previously reported as positive for the nuclear marker Ki67 [6,7]. However, it is important to note that Ki67 marks all cells outside G_0_. Thus, rather than pinpointing proliferating cells in the S-G_2_/M phases of the cell cycle, Ki67^+^ cells can reflect pre-activated G_1_ cells prior to DNA synthesis, or metabolically-active G_1_ cells that had previously proliferated in lymphoid organs. Indeed, when a DNA dye was applied to identify memory phenotype, peripheral blood CD8^+^ T cells in S-G_2_/M, less than 0.1% of cells stained positive [6]. Thus, there is *de facto* no data to support the assumption that the peripheral blood of subjects with active infection or immunopathology might carry T cells with residual signs of proliferation initiated in the draining lymph nodes. In part because of this, there is an almost universal perception that peripheral blood lymphocytes are resting passenger cells *en route* between proliferation in the lymphoid organs, effector function in extra-lymphoid tissues, and accumulation as memory cells in bone marrow and extra-lymphoid memory niches [6,[8], [9], [10], [11], [12]].

For four main reasons, this study has revisited this issue. First, we recently found that following inoculation with a recombinant viral vaccine, mice displayed antigen-specific, peripheral blood CD8^+^ T cells in S-G_2_/M within 10 days after priming, and within 3 days after boosting. A kinetics analysis post-boost showed that the actively cycling CD8^+^ T cells were sharply diminished by day 7 and had virtually disappeared by day 44 [13]. Second, detecting such cells relied on dual use of Ki67 plus a DNA stain, Hoechst 33342, and a relaxed lymphocyte gate [13], without which the high side scatter (SSC) of proliferating T cells(reflecting their mitochondrial and chromatin dynamics (ref. in Refs. [13]), and mTOR-induced metabolic changes [14]) risked their exclusion from commonly used lymphocyte flow cytometry gates. Because of this, previous Peripheral Blood Mononuclear Cell (PBMC) monitoring may have unwittingly underestimated proportions of antigen-specific T cells responding at early times following challenge. Third, any potential to gain insight into T cell dynamics by applying Ki67+Hoechst to peripheral blood could be broadly applicable in clinics without access to state-of-the-art “omics” capabilities. And fourth, any capacity to use the peripheral blood to draw inferences about events in tissue sites could overcome the challenges of longitudinally sampling human extra-lymphoid organs.

This study shows that T cells in peripheral blood are not all resting, passenger cells, but can include cells in S-G_2_/M phase, collectively termed “T Double S” (“T_DS_”) cells [“T cells in S-phase *in Sanguine*”]. “T_DS_” cells were reproducibly found to significantly different degrees in three scenarios: healthy donors (HD); subjects with Infectious Mononucleosis (IM); and T1D patients. In T1D patients, islet antigen-specific CD8^+^ T_DS_ frequencies were associated with activated effector functions. Thus, re-evaluating the conventional view of peripheral blood lymphocytes as resting cells in transit has extended the capacity of routine peripheral blood immuno-monitoring to track immunodynamics in diverse settings, including tissue-specific immunopathology.

## 2 Methods

### 2.1 Mouse study

The OT1 mouse study was performed in full compliance with the UK Home Office regulations (project license reference nb: 7009056).

### 2.2 Human study

Stored PBMC samples from individuals with active IM, patients with recent onset T1D and HD were included in this study. The T1D study was approved by the UK National Research Ethics Service (REC# 08/H0805/14), and the IM study by the South Birmingham Local Research Ethics Committee (14/WM/1254). Written informed consent was obtained from all participants prior to inclusion in the study.

For the IM study, 6 samples from HLA-A*02-positive donors were included: 4 Monospot positive IM donors, and 2 EBV carrier HD (Supplemental Table 1). For the T1D study, 21 samples from HLA-A*02-positive females were included: 11 samples from individuals with newly diagnosed T1D (mean age 26.1 years ±SD 5.5; mean disease duration after diagnosis 133.0 days ±SD 68.6) and 10 samples from age-matching HD (mean age 26.8 years ±SD 4.4) (Supplemental Table 2). T1D patients were not prescribed with steroids or other immunosuppressive medications, and they did not receive immunoglobulin treatments or blood products in the 3 months prior to blood withdrawal. For control of T_DS_ assay technical reproducibility, we used PBMC from a CMV carrier HLA-A*02-positive female HD.

### 2.3 Antigen-stimulation of OT1 cells and mitotracker incubation

OT1 C57BL6 SJL CD45.1 mice [15], bearing a transgenic T Cell Receptor (TCR) against OVA 257–264 in the context of H-2K^b^, were housed at the Crick Institute Biological Research Facility, according to Institutional guidelines. Mice were sacrificed by cervical dislocation and single cell suspensions were prepared from inguinal, axillary and mesenteric lymph nodes (LN). Cells were plated in RPMI 1640 medium with glutamine, 10% FCS, pen/strep and β mercaptoethanol in 6-well plates (2.5 × 10^7^/well), with or without OVA 257–264 peptide at 1 ng/ml. After 24 h-incubation at 37 °C with 5% CO_2_, cells were harvested, washed and re-plated in RPMI 1640 medium with 10 nM Mitotracker Deep Red (Invitrogen, Thermofisher Scientific, Waltham, MA, USA). After 20 min-incubation at 37 °C with 5% CO_2_, cells were collected and washed.

### 2.4 OT1 cell membrane staining

Mitotracker Deep Red-stained OT1 LN cells were incubated with eBioScience eFluor780 fixable viability dye (Invitrogen, Thermofisher Scientific) for live/dead cell discrimination, and washed. After blocking with FcBlock at 5 μg/ml for 15 min at 4 °C (Becton Dickinson, San Jose, CA, USA), cells were stained with anti-CD8β conjugated to phycoerythrin (PE) at 2 μg/ml for 15 min at 4 °C (clone YTS156.7.7, Biolegend, San Diego, CA, USA).

### 2.5 PBMC membrane staining

Heparinized blood samples were collected from either T1D patients and corresponding HD controls at Guy's Hospital of London, or IM patients following positive Monospot test at Queen Elizabeth Hospital Birmingham and corresponding HD controls at the University of Birmingham. PBMC were isolated by Ficoll gradient separation and immediately frozen, except for I-209, where B cells were depleted from the PBMCs first using CD19 Pan B Dynabeads (ThermoFisher Scientific). PBMCs were stored in liquid nitrogen for up to approximately 6 years in the T1D study, and 15 years in the IM study. After thawing, PBMC were either firstly stained with tetramers (for experiments with antigen-specific CD8^+^ T cells) and then with mAbs against membrane markers, or directly stained with anti-surface mAbs (for all the other experiments). Finally, live/dead cell staining was applied to all the samples.

The following allophycocyanin (APC)-conjugated peptide-loaded HLA-A*02 (pHLA) tetramers were used: PPI_15-24_, InsB_10-18_, GAD_114-123_, IGRP_265-273_, and IA-2_797-805_ HLA-A*02 tetramers (islet tetr); CMV pp65_495-503_ and EBV BMLF1_280-288_ HLA-A*02 tetramers (CE tetr), EBV BMLF1_280-288_ HLA-A*02 tetramers alone (EBV tetr), or CMV pp65_495-503_ HLA-A*02 tetramers alone (CMV tetr). pHLA were generated as previously described [16], see Supplemental Methods for more details. Tetramer and mAbs staining was performed as previously described [[16], [17], [18]] (Supplemental Methods for more details).

### 2.6 Intranuclear staining

Cells were fixed and permeabilized with Foxp3/Transcription Factor Staining Buffer (eBioscience, Thermofisher Scientific). Intracellular staining was performed at RT for 30 min with anti-Ki67 mAb (FITC-conjugated clone SolA-15 eBioscience at 5 μg/ml for mouse OT1 cells; Alexa Fluor 700-conjugated clone B56, Becton Dickinson for human PBMC), and anti-FoxP3 mAb (Alexa Fluor 647-conjugated clone 259D, Biolegend for human PBMC, where indicated). After washing, cells were incubated in PBS with 2 μg/mL Hoechst 33342 (Thermofisher Scientific) at RT for 15 min. After centrifugation at 400*g*, cells were resuspended in PBS [13].

### 2.7 Imaging flow cytometry analysis

Samples were acquired on an Image Stream^X^ Mk II (Amnis, Luminex Corporation, Austin, TX, USA) using INSPIRE software. Data were analysed using IDEAS software.

### 2.8 Flow cytometry analysis

Samples were acquired on an LSRFortessa flow cytometer (Becton Dickinson) using DIVA software. Data were analysed using FlowJo software, v.10 (FlowJo, Ashland, OR, USA).

### 2.9 Nanostring gene expression analysis

The multiplexed NanoString nCounter™CAR-T Characterization panel was used as expression assay for profiling 780 human genes (NanoString Technologies, Inc., Seattle, WA, USA). The assay was performed according to the manufacturer's protocol (see Supplemental Methods for more details). Pre-processing and normalization of the raw counts was performed with nSolver Analysis Software v4.0 (www.nanostring.com). Gene expression data were normalized by using all the 10 housekeeping genes present in the CAR-T Characterization panel. The 6 spiked-in RNA Positive Control and the 8 Negative controls present in the panel were used to confirm the quality of the run. The heatmap was generated using the package “ComplexHeatmap” within R version 3.5.1. Prior to clustering, data were log10 transformed and mean centered and genes related to TCR were removed from the data. The heatmap data was clustered by selecting “clustering_distance_columns = canberra” and “clustering_method_columns = ward.D" with all the rest of parameters left as default [19,20].

### 2.10 Statistics

Data represent individual samples, mean (bars) ± SEM (error bars). Two-tailed Mann-Whitney U (two groups) and Kruskal-Wallis with Dunn's multiple comparison (more than two groups) statistical tests were used to compare different donor groups. Two-tailed Wilcoxon matched pairs signed rank (two groups) and Friedman with Dunn's multiple comparison (more than two groups) statistical tests were used to compare multiple T cell subsets from the same donors. Fisher's exact test was used to compare number of “T_DS_^+^^”^ subjects between HD and T1D. *P* values were considered significant when *P < 0.05; **P < 0.01; ***P < 0.001; ****P < 0.0001. Statistical analysis was performed using Prism v.6.0f, GraphPad Software (La Jolla, CA, USA).

## 3 Results

### 3.1 Properties of proliferating CD8^+^ T cells

The initial foundation for this study was the identification of an activated T cell phenotype in the blood of mice 10 days post priming or 3 days post boosting [13]. First we investigated whether this was a close phenocopy of T cells responding to antigen-specific stimulation. Thus, we exposed mouse OT1 LN T cells to cognate peptide-MHC (pMHC) antigen and undertook Imaging flow cytometry (ImageStream^X^ MK II platform).

Our gating strategy (Supplemental Fig. 1A) accommodated the fact that so-called SpeedBeads used to capture images in focus occasionally coincide with cells, resulting in high SSC intensity values emanating from a localised spot. This is in contrast to *bona fide* cycling cells for which high SSC emanates from across the image (Supplemental Fig. 1B). A so-called SSC Bright Detail Intensity gate excluded such artefacts (Supplemental Fig. 1A, Gate 5; “without SpeedBeads”). Likewise, Gate 6 excluded “shadow doublets” in which some Ki67^lo^ cells scored artefactually high for DNA content because two cells sat almost directly in front of one other by contrast to *bona fide* singlet cells, which additionally stained for a mitochondrial tracker (Supplemental Figs. 1A, C, D).

**Fig. 1:**
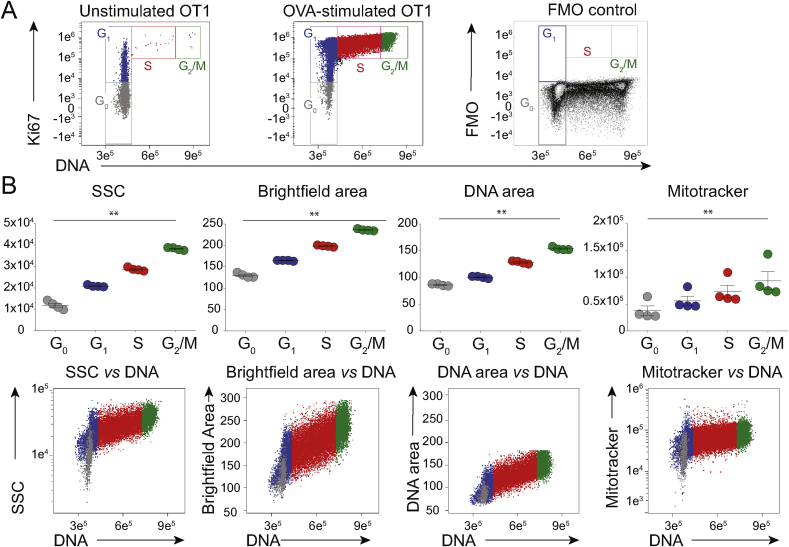
OT-I CD8^+^ T cells have increased signal for SSC, DNA and mitochondria as they cycle in response to antigen. OT1 LN cells were stimulated with antigen *in vitro*, and analysed by ImageStream. **A)** DNA/Ki67 flow cytometry plots of OT1 LN CD8^+^ cells either unstimulated (left), or stimulated with OVA 257–264 peptide (centre), with Ki67 FMO plot of stimulated CD8^+^ cells (right). Gates for G_0_, G_1_, S and G_2_/M are indicated. **B)** Median SSC (arbitrary units), Brightfield (μm^2^), and DNA image area (μm^2^), and Mitotracker Intensity (arbitrary units) of CD8^+^ cells in G_0_, G_1_, S and G_2_/M, gated as in A (top, N = 4 experiments). Bivariate DNA intensity (arbitrary units) plots from a representative experiment (bottom, colour code as in A). Statistical analysis performed using Friedman's test with Dunn's multiple comparison tests.

Employing this strategy, most unstimulated OT1 T cells were Ki67^(−)^Hoechst^lo^ (i.e. G_0_ cells) (Fig. 1A, left; colour-coded grey), whereas antigen-stimulated cells collectively displayed a signature profile of cells in G_1_ (Ki67^+^Hoechst^lo^; blue), S (Ki67^+^Hoechst^int^; red), and G_2_/M (Ki67^+^Hoechst^hi^; green) (Fig. 1A, centre panel; see Supplemental Fig. 2A for summary of four experiments). Note that Ki67 staining specificity was evident from an FMO (fluorescence minus one) control (Fig. 1A, right-hand panel). Thus, the phenotype of cells identified in the blood of vaccinated mice [13] was equivalent to that of *in vitro* antigen-stimulated OT1 T cells.

To characterize those cells further, the colour-coded populations were assessed by ImageStream, and found in order of cell-cycle progression to display precisely quantitative increases in SSC; mitotracker uptake; brightfield, as a measure of cell size; and DNA image area, as a measure of nuclear size (Fig. 1B, top; Supplemental Fig. 2B for illustrative images). The segregation of discrete cell-cycle phases with brightfield image area, nuclear size, and SSC was particularly evident, and parameters clearly correlated when cross-compared (Fig. 1B, bottom). These findings provided platform for revisiting the presence of cycling T cells in human peripheral blood.

### 3.2 Detection of cycling human peripheral blood CD8^+^ T cells

**Fig. 2:**
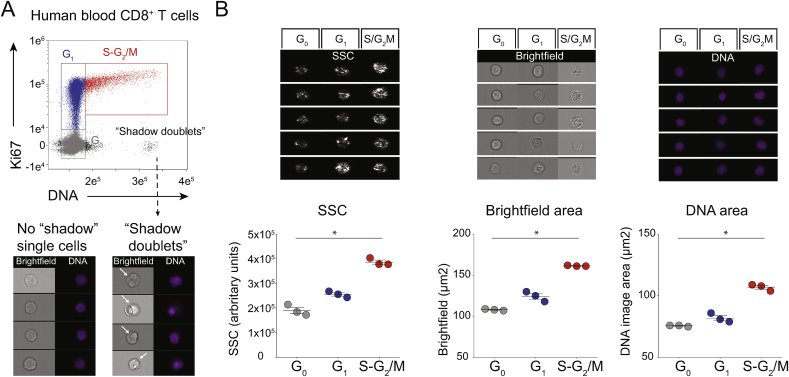
Human blood CD8^+^ T cells have increased signal for SSC and DNA as they progress through the cell cycle. CD3^+^CD8^+^ cells from HD PBMC were analysed by ImageStream. **A)** DNA/Ki67 plot (top), showing “no shadow” single cells in G_0_ (grey), G_1_ (blue) and S-G_2_/M (red), with assigned colour codes adopted in all subsequent analyses, and Ki67^(−)^Hoechst^hi^ “shadow doublets”. Brightfield and DNA images showing “no shadow” single cells (bottom left) and “shadow doublets” (arrows, bottom right). **B)** Examples (top) and summary of results (lower, N = 3 HD) for median SSC intensity (arbitrary units), Brightfield (μm^2^) and DNA Image Area (μm^2^) of single cells in G_0_, G_1_, and S-G_2_/M, gated as in A and Supplemental Fig. 3A, Step 1 to 6 (bottom, N = 2 experiments with a total of 3 HD). Statistical analysis performed using Friedman's test with Dunn's multiple comparison tests.

Given the demonstration that our gating strategy could provide multiple quantitative discriminators of antigen-activated T cells, we employed a similar gating strategy (see Supplemental Fig. 3A) to investigate the peripheral blood of three HD. By comparison to mouse cells analysed by either ImageStream (Supplemental Fig. 1) or flow cytometry [13], there was always a higher incidence of Ki67^(−)^Hoechst^hi^ cells (Fig. 2A, “shadow doublets”), starkly illustrating the limitations of Hoechst content as a sole criterion for analysing human PBMC. Nonetheless, with “shadow doublets” excluded (Supplemental Fig. 3A, Step 6), we could identify CD8^+^ T cells that phenocopied the discrete subsets of OT1 T cells: namely, Ki67^(−)^Hoechst^lo^ (G_0_), Ki67^+^Hoechst^lo^ (G_1_), and Ki67^+^Hoechst^int/hi^ (S-G_2_/M) (Fig. 2B, Supplemental Fig. 3B). We define those blood T cells that are in S-G_2_/M as “T_DS_” cells. Parenthetically, T cell cycle stages showed completely comparable enumeration between fresh and frozen aliquots of identical PBMC, and “shadow doublets” were present in both (data not shown). Hence, for reasons of broad applicability, our study was performed with frozen PBMC.

### 3.3 Multiple subtypes of proliferative PBMC T cells

**Fig. 3:**
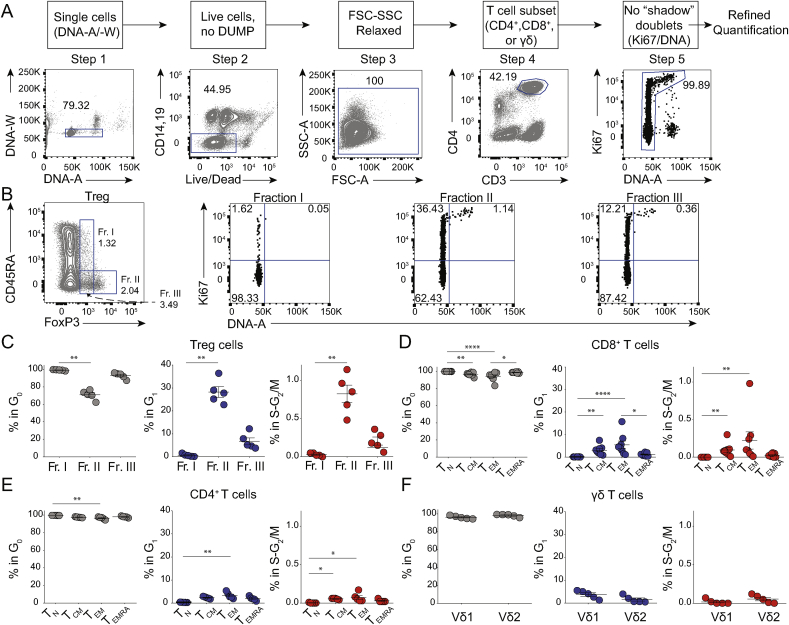
Some T cells in HD PBMC are in S-G_2_/M phases. **A)** Gating strategy (top) and example of CD4^+^ T cell flow cytometry analysis (lower). **B)** Refined quantification of T_reg_ Fractions I, II and III among CD4^+^ T cells gated as in A, Step 1 to 5, (left), with illustrative examples of cells in G_0_, G_1_, and S-G_2_/M as assessed by Ki67/DNA profiles. Numbers represent cell percentages in the indicated gate. **C–F)** summary of refined cell cycle analysis of T_reg_ Fractions (C), of T_N_, T_CM_, T_EM_ and T_EMRA_ among either CD8^+^ (D), or conventional CD4^+^ T cells (E), and of Vδ1 and Vδ2 among γδ T cells (F). (N = 7 experiments with a total of 12 HD). Statistical analysis performed using Friedman's test with Dunn's multiple comparison tests (C-E), and Wilcoxon test (F).

We next examined by flow cytometry whether T_DS_ cells were identifiable for other T cell subsets. Prior to gating on discrete T cell phenotypes, we used a “relaxed” lymphocyte gate (Fig. 3A, Step 3) so as to include cells with high SSC [13,14]. Thereafter, step 5 excluded “shadow doublets”, which were usually ~0.3%, and that were present even when a DNA-A/DNA-W gate was employed for single cell discrimination at Step 1 (Fig. 3A; Supplemental Fig. 4A, C, E).

Applying this approach to three phenotypic subsets of regulatory T (T_reg_) cells, we confirmed that Fraction I cells were largely quiescent (~98% Ki67^(−)^Hoechst^lo^) [21], while 20%–40% of Fraction II cells were in G_1_, and 0.5%–1.5% in S-G_2_/M, whereas Fraction III cells displayed an intermediate phenotype (Fig. 3B and C). Thus, peripheral blood harbours proliferating T_reg_ cells, particularly in Fraction II.

Likewise, when applying this approach to defined subsets of naïve (T_N_) and effector and memory CD8^+^ and CD4^+^ T cells (Supplemental Fig. 4A–D), we found that central-memory (T_CM_) and effector-memory (T_EM_) CD8^+^ T cells included significantly more cells in G_1_ than did T_N_ cells. Moreover, a similar pattern was observed for CD4^+^ T_EM_ cells (Fig. 3D and E; Supplemental Fig. 4A–D). This notwithstanding, among all conventional T cell subsets —particularly CD4^+^ T cells— T_DS_ cells were rare and sometimes entirely absent, contrasting with their invariable presence among T_reg_ Fraction II cells. Furthermore, γδ T cells showed very low T_DS_ cell representation, for either the predominant peripheral blood Vδ2^+^ subtype or the Vδ1^+^ subtype most commonly associated with extra-lymphoid tissues (Fig. 3F; Supplemental Fig. 4E and F).

### 3.4 T_DS_ cells associate with clinical status

**Fig. 4:**
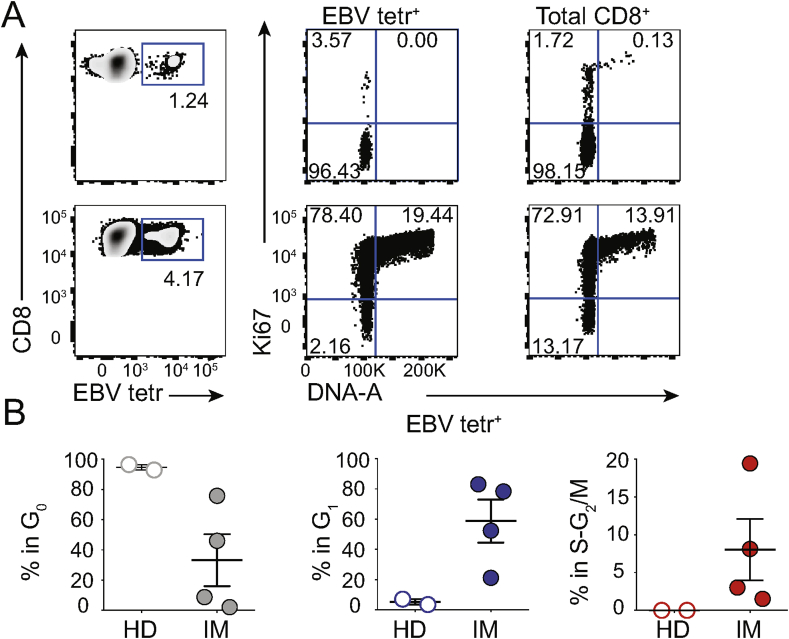
T_DS_ cells in Infectious Mononucleosis (IM) patients. **A)** Illustrative flow cytometry EBV tetr/CD8 plot (left); with Ki67/DNA plots for EBV tetr^+^ (centre) and total CD8^+^ T cells (right) from HD (top) and IM patients (bottom). **B)** Cell cycle summary in IM identifies T_DS_ cells in EBV tetr^+^ cells (right hand panel)**.** (N = 1 experiment with a total of 2 HD and 4 IM).

To determine whether T_DS_ cell representation might be informative *vis-a-vis* ongoing infection, we applied the strategy shown in Supplemental Fig. 4C to examine HLA-A*02-restricted CD8^+^ T cells reactive to a single immunodominant Epstein Barr virus peptide (EBV BMLF1_280-288_), as detected *via* multimeric peptide-HLA (pHLA) tetramers (“EBV tetr”) [22]. Illustrative data (Fig. 4A; upper panels) show an EBV-carrying HD in whom 1.24% of CD8^+^ PBMC were EBV tetr^+^, of which 3–4% were in G_1_ and none was in S-G_2_/M. Likewise, >98% of total CD8 T cells were in G_0_ (Fig. 4A; top, right hand panel). Conversely, for a donor experiencing IM, a profound clinical manifestation of primary EBV infection (see Supplemental Table 1), EBV tetr^+^ cells comprised only ~4% of CD8^+^ PBMC (i.e. <4-fold increase over the HD), but >75% of these were in G_1_ and ~20% in S-G_2_/M. Moreover, >85% of total CD8 T cells were in G_1_-S-G_2_/M (Fig. 4A; lower panels).

Importantly, high frequencies of EBV tetr^+^ T_DS_ cells and of total CD8^+^ T_DS_ cells were seen in each of four IM patients and they readily distinguished each individual patient from either of 2 HDs. By contrast, frequency of blood-borne EBV tetr^+^ cells was a less compelling discriminator (Fig. 4B; Supplemental Fig. 5A and B). The high frequency of T_DS_ cells among total CD8 T cells of IM patients most likely reflects antigen-specific expansion of T cells specific for EBV antigens other than BMLF1_280-288_-HLA-A*02, with minor degrees of bystander stimulation [23,24].

### 3.5 T_DS_ cells in autoimmune disease

**Fig. 5:**
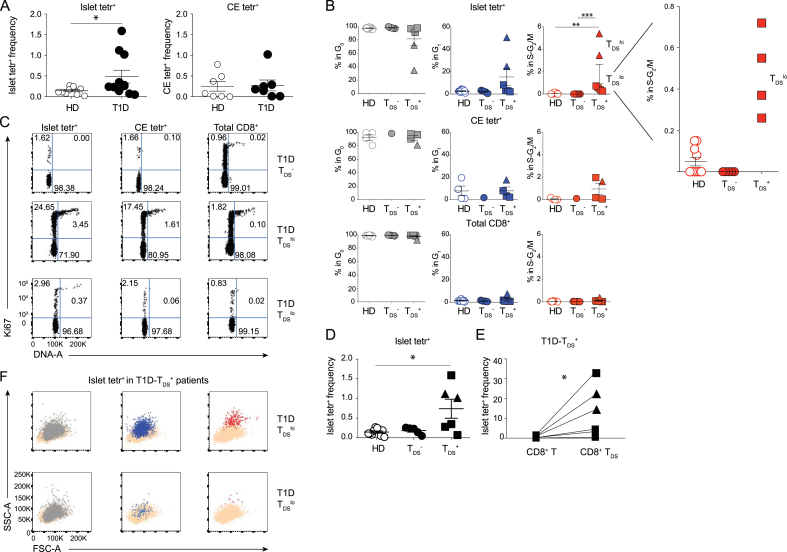
T_DS_ cells in Type 1 Diabetes (T1D) patients. **A)** Frequency of islet tetr^+^ (left) and CMV + EBV (CE) tetr^+^ cells (right) among total CD8^+^ T cells in HD and T1D patients. **B)** Summary of cell cycle data of islet tetr^+^, CE tetr^+^, and total CD8^+^ T cells in HD, T_DS_^−^ and T_DS_^+^ T1D patients [triangles, T_DS_^hi^; squares, T_DS_^lo^]. **C)** Examples of Ki67/DNA plots of islet tetr^+^ (left), CE tetr^+^ (centre), and total CD8^+^ T cells (right) from T_DS_^−^ (top), T_DS_^hi^ (middle) and T_DS_^lo^ (bottom) T1D patients. **D)** Frequency of islet tetr^+^ cells among total CD8^+^ T cells in HD, T1D-T_DS_^-^, and T1D-T_DS_^+^. **E)** Frequency of islet tetr^+^ cells in T1D-T_DS_^+^ patients, calculated either among total CD8^+^ T cells or among CD8^+^ T_DS_ cells. **F)** Illustrative FSC-A/SSC-A plots of T1D-T_DS_^hi^ (top) and T1D-T_DS_^lo^ (bottom) islet tetr^+^ cells in G_0_ (left), G_1_ (centre) and S-G_2_/M (right), overlaid on total CD8 T cells. Statistical analysis performed using Mann-Whitney *U* test (A), Kruskal-Wallis tests with Dunn's multiple comparison (B, D), and Wilcoxon test (E). (N = 6 experiments with a total of 10 HD and 11 T1D).

T1D patients harbour CD8^+^ T cells reactive to pancreatic islet autoantigens. Using a mixture of tetrameric islet antigen pHLA-A*02 complexes (“islet tetr”) [16], we screened PBMC from 11 female T1D patients within 1 year from diagnosis, and 10 age-matched female HDs (Supplemental Table 2). Whereas our cohort of T1D patients considered as a group had significantly more CD8^+^ T cells reactive to pancreatic islet autoantigens than matched HD (Fig. 5A, left panel), this parameter could not distinguish most patients, either from one another or from healthy controls (Supplemental Fig. 6A, and Fig. 5A, left panel) [16,25]. There was likewise no discriminating trend in the frequencies of CD8^+^ T cells reactive to tetramers comprising a mixture of EBV and CMV peptide antigens (“CE tetr”), that usually reflect a carrier state for one or both viruses (Supplemental Fig. 6A, and Fig. 5A, right panel).

By contrast, our analysis of T_DS_ frequency among islet tetr^+^ CD8^+^ T cells identified two groups: “T_DS_^−^” patients displaying ~0% of T_DS_ cells, and “T_DS_^+^” patients displaying up to ~5% T_DS_ cells (Fig. 5B–C). To establish a firm basis for this classification, we considered that the mean frequency of islet tetr^+^ CD8^+^ T_DS_ cells across the HD cohort was 0.049% (SD = 0.066%) (Fig. 5B; top row, righthand panel and insert), and assigned T_DS_^+^ classification to those individuals whose islet tetr^+^ T_DS_ representation exceeded HD mean + 3xSD (i.e. >0.248%). This T_DS_^+^-based classification rubric delineated a highly significant difference between T1D patients (6/11) *versus* HD (0/10) (*p* = 0.0124 Fisher's exact test) (Fig. 5B; top row, righthand panel). Moreover, T_DS_^+^ patients showed a significant difference in the frequencies of islet tetr^+^ cells versus HDs whereas this was not so for T_DS_^−^ patients (Fig. 5D). By contrast the distinction between T_DS_^+^ and T_DS_^−^ T1D patients was not attributable to differences in the duration of PBMC storage (mean storage time and SD: 1.80 years ±2.08, and 2.14 years ±2.02, respectively; *p* = 0.6623 Mann-Whitney test). Additionally, so that we might further understand the underlying basis of the T_DS_^+^-based classification, we used triangles to denote “T_DS_^hi”^ patients with highly proliferative islet tetr^+^ CD8^+^ T cells (>3% in S-G_2_/M), and squares for those T_DS_^+^-patients showing lower proliferation (T_DS_^lo^) (Fig. 5B–C). When this criterion was applied, we noted that it did not correlate directly with the increased representation of islet tetr^+^ T cells, since the highest value was in a T_DS_^lo^ patient (Fig. 5D, square). However, a potential and profound significance of T_DS_^hi^ *versus* T_DS_^lo^ cells is considered later in this study when transcriptomic analyses are reported.

We also evaluated total CD8^+^ T_DS_ cells in all individuals and CE tetr^+^ T_DS_ cells in a subset of donors in which a carrier state for one or both viruses was reflected in a frequency >0.01% of CE tetr^+^ cells. For some T_DS_^+^ patients, CE tetr^+^ T_DS_ cells were evident, but for others they were not, possibly related to observations that the activation of CMV-specific and/or EBV-specific T cells is very variable in different clinical settings [26]. Conspicuously, the T_DS_ fraction among total CD8^+^ T cells was extremely low in every case, including T_DS_^hi^ subjects (Fig. 5B–C). Focusing only on those donors for which CE tetr^+^ cells could be evaluated, a difference in the frequency of T_DS_ cells between T_DS_^+^ patients (N = 4) and HD (N = 4) was found among islet tetr^+^ T cells (*p* = 0.0286, Mann-Whitney test), but not among CE tetr^+^ cells (*p* = 0.0857), or total CD8^+^ T cells (*p* = 0.2000). Hence, the segregation of T1D patients according to T_DS_ status was related to auto-antigen specific cells rather than either total or virus-specific T cells. Indeed, islet antigen-specific T cells were significantly enriched among CD8^+^ T_DS_ cells relative to total CD8^+^ T cells (Fig. 5E). It was again evident that the high SSC of islet tetr^+^ T_DS_ cells placed many of them on the very edge of the CD8^+^ T cell gate (Fig. 5F, colour-code red), risking their exclusion by conventional lymphocytometry. Of note, confidence in the assessment of subject-specific representation of cells in G_1_ and S-G_2_/M was provided by batch effect controls using a single donor's PBMC on each assay run and quantifying for the same specificity, namely CMV (Supplemental Fig. 6B).

### 3.6 T1D patients with high T_DS_ cells have a distinct effector signature

**Fig. 6:**
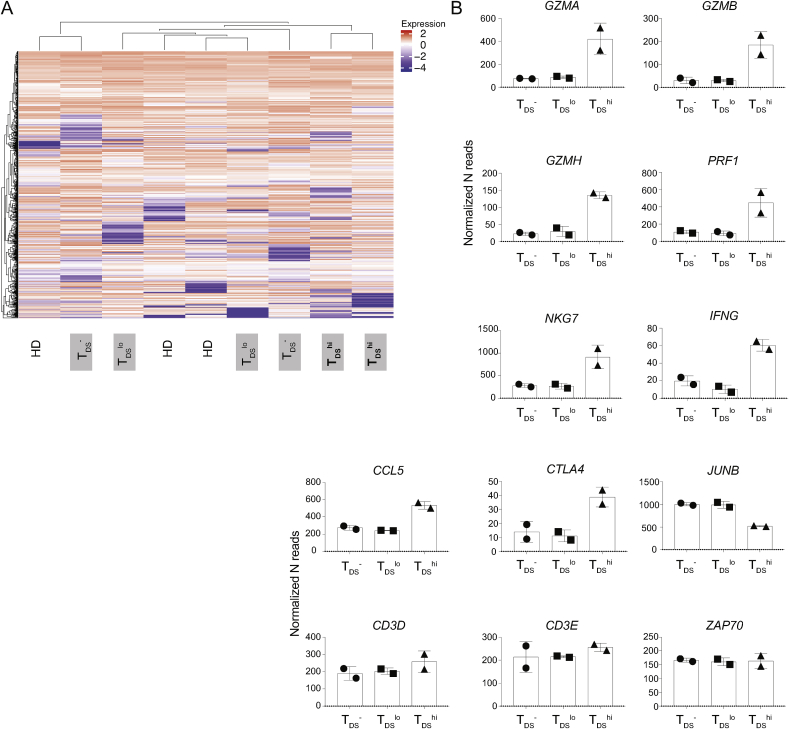
Islet-specific CD8 T cells from T1D-T_DS_^hi^ patients display activated effector potentals Islet tetr^+^ CD8^+^ T cells were FACS-sorted from 3 HD, 2 T1D-T_DS_^-^, 2 T1D-T_DS_^lo^, and 2 T1D-T_DS_^hi^ donors. Nanostring analysis was performed using commercially available CAR-T gene panel. **A)** Hierarchical clustering was performed using R. **B)** Representative effector signature genes expressed by T1D-T_DS_^-^, T1D-T_DS_^hi^ and T1D-T_DS_^lo^, i.e. *GZMA, GZMB, GZMH, PRF1, NKG7, IFNG* and *CCL5*. Analysis of *CTLA4*, *JUNB*, and *CD3D, CD3E* and *ZAP70* is included.

To better understand the functional implications of T_DS_ assay in T1D patients, Nanostring gene expression analyses were undertaken for islet tetr^+^ CD8^+^ T cells purified from: two T1D patients bearing highly proliferative [T_DS_^hi^] cells (see above), two donors bearing T_DS_^lo^ cells; two T_DS_^−^ donors; and 3 HDs. Unsupervised analysis co-clustered the 2 donors bearing T_DS_^hi^ samples away from all the others (Fig. 6A).

Strikingly, the 2 donors bearing T_DS_^hi^ cells were clearly enriched in transcripts encoding: cytolytic effector molecules, including Granzymes A, B, and H, and perforin and NKG7 which have been shown to reflect T cell effector maturation; IFNγ and CCL5 (RANTES), two secreted effectors of cytolytic T cells; and CTLA-4 whose upregulation reflects TCR-mediated T cell activation (Fig. 6B). In parallel, *JUNB*, whose downregulation is associated with movement into cell-cycling, was expressed less by T_DS_^hi^ donors (Fig. 6B), while other genes, including those encoding CD3 proteins and associated signaling molecules, were comparably expressed (Fig. 6B). The transcripts of genes typically expressed by “exhausted” T cells in chronic responses, e.g. *PDCD1* (*PD-1)* [27,28], were extremely low and comparable across the T_DS_^hi^, T_DS_^lo^, and T_DS_^−^ donors (Supplemental Table 3). Additionally, the expression of genes associated with CD8^+^ T_reg_ cells, e.g. *IKZF2* (*HELIOS*) [29,30] was low and not up-regulated in highly proliferative donors (Supplemental Table 3). Hence, the otherwise unbiased discrimination of patients by high T_DS_ levels effectively identified patients whose peripheral blood contained T cells in a highly activated, effector state.

## 4 Discussion

We have developed a means for the reliable, highly sensitive identification in peripheral blood of proliferating T cells, as judged by their being in the S-G_2_/M phases of the cell-cycle, which we termed “T_DS_” cells. Although our focus was on CD8^+^ T cells, T_DS_ cells could be found in other compartments, particularly Fraction II T_reg_ cells. Only a few T_DS_ were detected among CD4^+^ T_EM_ and almost none among CD4^+^ T_CM_ cells, while T_DS_ were more common among corresponding CD8^+^ T cell subsets. This may be consistent with the reported dependence of antigen-stimulated CD4^+^ T cell proliferation on more prolonged antigen presentation [31,32].

A clinical association of T_DS_ cells was suggested by their abundance in IM, set against a backdrop of activated T cells. Conversely, the inter-individual variation in levels of islet-specific CD8^+^ T_DS_ cells among T1D patients appeared to mostly reflect discrete specific responses to pancreatic autoantigens. Given that the T1D patients were all relatively recently diagnosed (Supplemental Table 2), the future opportunity will exist to test whether T_DS_ cells will correlate with some aspects of disease progression, particularly given that high T_DS_ cell numbers clearly predicted the presence in circulation of islet-reactive CD8^+^ T cells with high effector potentials. We note that while, in the setting of T1D, lower T_DS_ cell numbers, as seen in T_DS_^lo^ patients, were not associated with heightened activated effector signatures, they did contribute to the significant difference in the frequency of islet antigen-reactive cells between T1D patients as a whole and HD.

Of note, the capacity to distinguish individuals based on the cell-cycle status of peripheral blood CD8^+^ T cells reactive to islet antigens, strongly suggests that T_DS_ can provide windows onto the dynamics of immunological processes within tissues and their draining LNs, wherein islet antigens would be encountered. We therefore advocate incorporating T_DS_ analyses into immuno-monitoring in many settings, including site-specific autoimmunity, cancer, and allergy, in which regard, our standard operating procedures should be widely accessible.

Finally, the invariant occurrence of T_DS_ cells among Fraction II T_reg_ cells suggested ongoing suppressive regulation [21]. By contrast, T_DS_ cells were very rare among peripheral blood γδ T cells. Hence, T_DS_ could provide a sensitive means for analysing settings in which γδ T cells become activated. Given that γδ T cells are strongly implicated in lymphoid stress-surveillance [33], their monitoring could conceivably contribute to the early detection of disease occurrence and/or recurrence.

## Declaration of competing interest

ACH is a co-founder and equity holder in ImmunoQure, AG; Gamma Delta Therapeutics, and Adaptate Biotherapeutics; MP is employed by Sanofi.
