# Supplementary material for: Tracking immunodynamics by identification of S-G2/M-phase T cells in human peripheral blood
Source: J Autoimmun. 2020 Aug;112:102466. doi: 10.1016/j.jaut.2020.102466 (PMC7527781; doi:10.1016/j.jaut.2020.102466)
Supplement: Multimedia component 1 [file mmc1.pdf]

Supporting information to

Miguel Munoz Ruiz *et al*

"Tracking immunodynamics by identification of S-G_2/M-phase T cells in human peripheral blood"

Journal of Autoimmunity (2020), https://doi.org/10.1016/j.jaut.2020.102466

Supplemental methods

Tetramer assembly

Allophycocyanin (APC)-conjugated peptide-loaded HLA-A*02 (pHLA) tetramers were generated as previously described [34]. Tetramers were assembled over five successive additions of streptavidin-APC (Life Technologies, Thermofisher Scientific), in 20-minute steps, to monomeric pHLA at a molar streptavidin/pHLA ratio of 1:4. PBS was added to give a final multimer concentration of 0.1mg/ml pHLA content. The following tetramers were pooled and used to track islet-tetr$^+$ CD8 T cells: PPI_{15-24}, $InsB_{10-18}$, $GAD_{114-123}$, $IGRP_{265-273}$, and IA-$2_{797-805}$ HLA-A*02 tetramers (islet tetr) [16]. Previous work showed that differences in CD8 T cell differentiation/memory status between T1D patients and HD were similar across the 5 specificities [16]. We also used CMV $pp65_{495-503}$ plus EBV $BMLF1_{280-288}$ HLA-A*02 tetramers (CE tetr), EBV $BMLF1_{280-288}$ HLA-A*02 tetramers alone (EBV tetr), or CMV $pp65_{495-503}$ HLA-A*02 tetramers alone (CMV tetr). Tetramers were pooled according to islet or viral (CE) groups at 1µg/specificity/test, stored in the dark at 4°C and used on the same day as assembly.

PBMC membrane staining

For tetramer staining, PBMC were incubated for 20 minutes at 37°C with 5% CO_2 with the protein kinase inhibitor dasatinib (Axon Medchem, Reston, VA, USA) at a final concentration of 50nM to enhance the detection of low avidity T cells [16, 17], and with anti-CCR7 PE mAb (clone 150503, R&D System, Minneapolis, MN, USA). Then pHLA tetramers were added and cells were incubated for further 10 minutes at 37°C with 5% CO_2. After washings, unconjugated anti-APC mAb (clone APC003, Biolegend) at a final concentration of 10μg/ml was added, and cells were incubated on ice for 20 minutes to stabilize binding of APC-labeled tetramers [18].

For membrane staining the following mAbs were used: anti-CCR7 PE mAb, anti-CD3 PercpCy5.5 (clone SK7, Biolegend), anti-CD8 PE or PECy7 (clone SK1, Biolegend), anti-CD45RA PE CF594 (clone HI100, Becton Dickinson), anti-CD4 FITC (clone SK3, Becton Dickinson), anti-CD14 FITC (clone MP9, Becton Dickinson) and anti-CD19 FITC (clone HIB19, Biolegend), anti-TCR γδ PE-Cy7 (clone IMMU510, Beckman Coulter), anti-Vδ1 APC (clone REA173, Miltenyi Biotec), anti-Vδ2 PE (clone 123R3, Miltenyi Biotec).

Live/dead cell staining was performed with eBioScience eFluor780 fixable viability dye.

Nanostring gene expression analysis

The multiplexed NanoString nCounter™CAR-T Characterization panel was used as expression assay for profiling 780 human genes (NanoString Technologies, Inc., Seattle, WA, USA). The assay was performed according to manufacturer's protocol. In brief, FACS-isolated islet $tetr^+$ $CD8^+$ cells (at FACSAria Fusion-Becton Dickinson) were lysed in 2μl of RLT plus lysis buffer (Qiagen Plus) at 1,500 – 2,500 cells/sample and crude cell lysate was used as input material. Samples were spun at 400g for 5 minutes at 4°C, and finally snap-frozen on dry ice and stored at –80°C. mRNA expression was measured on NanoString

nCounter™ MAX system in a final volume of 15µl by using 2µl cell lysates mixed with a 3′ biotinylated Capture Probe and a 5′ Reporter Probe tagged with a fluorescent barcode of the CAR-T panel. Probes and target transcripts were hybridized at 65°C for 22 hours following manufacturer recommendations.

Hybridized samples were run for a total of three hours on the NanoString nCounter™ Preparation Station using the high-sensitivity protocol, in which excess Capture and Reporter Probes were removed and probe-transcript complexes were immobilized on a streptavidin-coated cartridge. The samples were then scanned at maximum scan resolution (555 fields of view) to count the individual barcodes on the nCounter™ Digital Analyzer (NanoString Techonologies), as described by the manufacturer.

Supplemental references

34. L. Wooldridge, H.A. van den Berg, M. Glick, E. Gostick, B. Laugel, S.L. Hutchinson et al. Interaction between the CD8 coreceptor and major histocompatibility complex class I stabilizes T cell receptor-antigen complexes at the cell surface. J Biol Chem 280 (2005) 27491-501. https://10.1074/jbc.M500555200

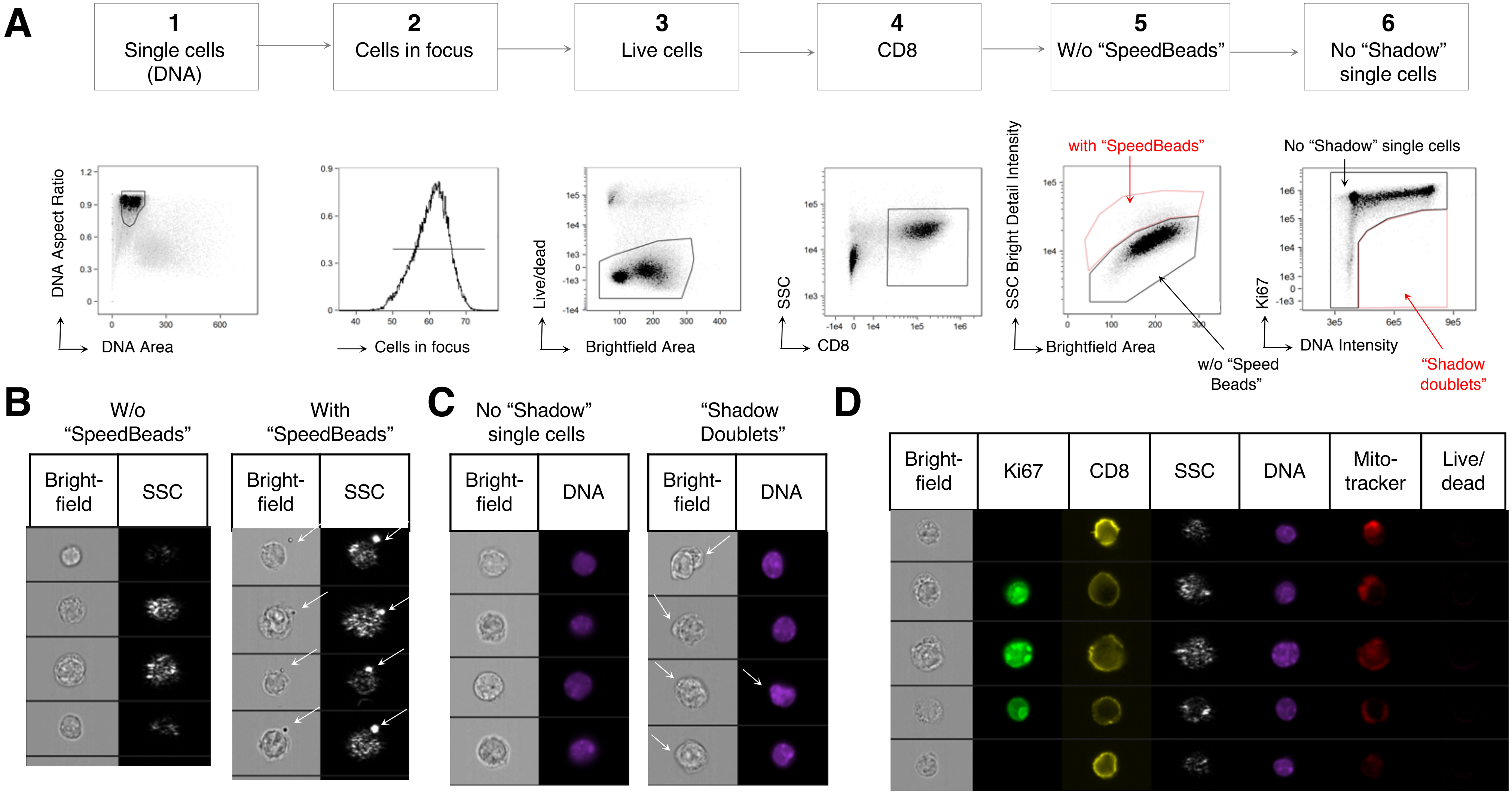

Supplemental Figure 1. Gating strategy for ImageStream analysis of *in vitro* stimulated OT1 cells and examples of images.
A) Scheme of the gating strategy (top), and example (bottom) of ImageStream analysis of OT1 cells after 24 hour-stimulation with OVA 257-264 peptide. The following cells were gated in 6 steps (black framed gates): single cells (Step 1); cells in focus (Step 2); live cells (Step 3); CD8$^+$ cells (Step 4); cells without (w/o) “SpeedBeads” (Step 5); no “shadow” single cells (Step 6). We specifically designed the gates at Step 5 and 6 for a refined quantitative analysis of single cells, by excluding “SpeedBeads” and “shadow doublets” (excluded events in red framed gates). **B)** Examples of brightfield and SSC cell images w/o (left) and with (arrow, right) “SpeedBeads”. **C)** Examples of brightfield and DNA images showing “no shadow” single cells (left) and “shadow doublets” (arrows, right). **D)** Examples of images showing brightfield, Ki67, CD8, SSC, DNA, mitotracker, and Live/dead staining of single CD8$^+$ cells, gated as in A (Step 1 to 6).

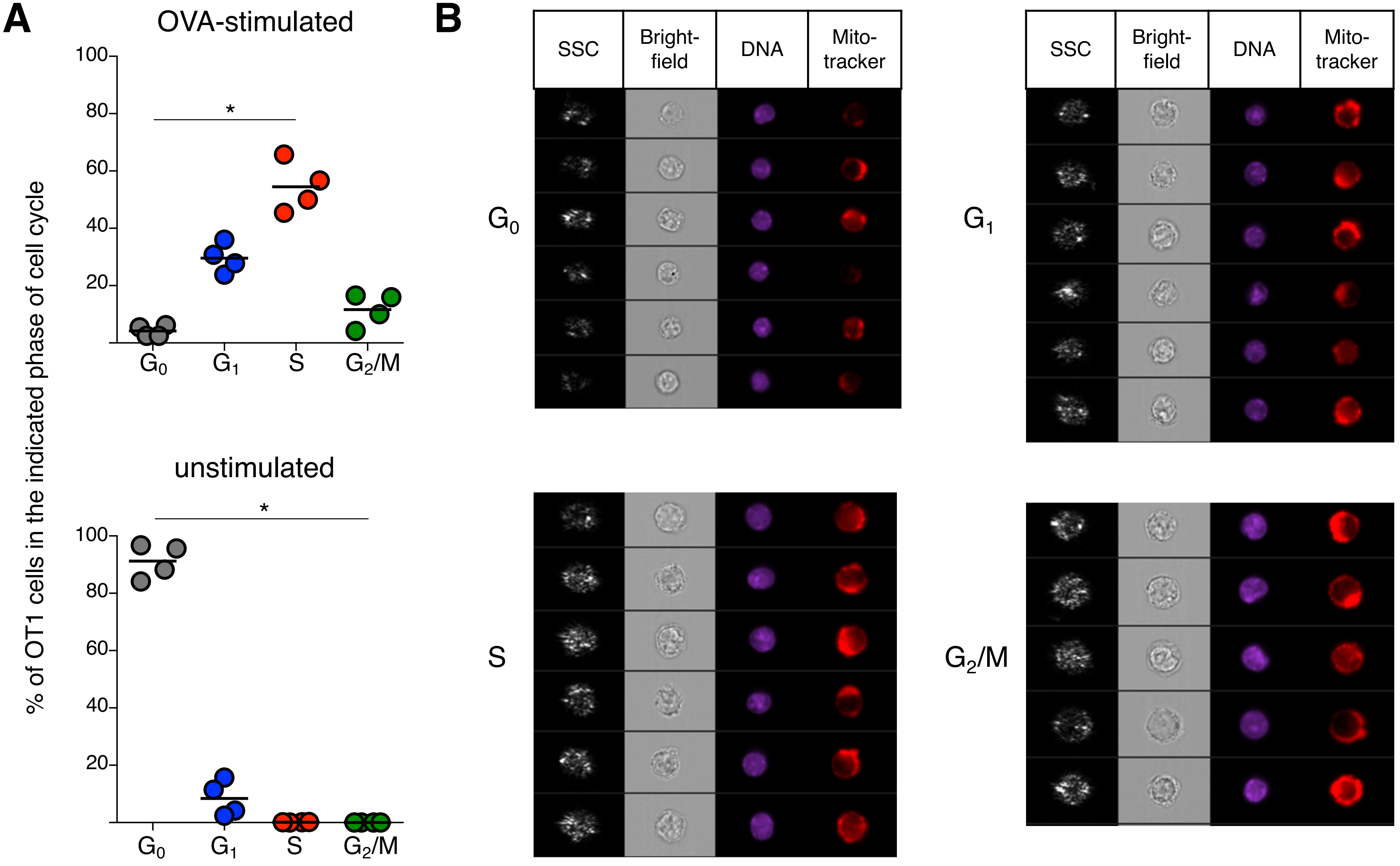

Supplemental Figure 2. ImageStream analysis of cycling antigen-stimulated OT1 cells.

A) OT1 LN cells were either stimulated or not with OVA 257-264 peptide *in vitro*, and the percentages of $CD8^+$ cells in G_0, G_1, S and G_2/M were determined by ImageStream, after gating as in Figure 1A (top, OVA peptide-stimulated cells; bottom, unstimulated cells cultured with medium alone; N=4 experiments). **B)** Examples of images showing SSC, brightfield, DNA and mitotracker staining of single $CD8^+$ cells in G_0 (top left), G_1 (top right), S (bottom left) and G_2/M (bottom right), gated as in Figure 1A. Statistical analysis performed using Friedman's test with Dunn's multiple comparison tests (A).

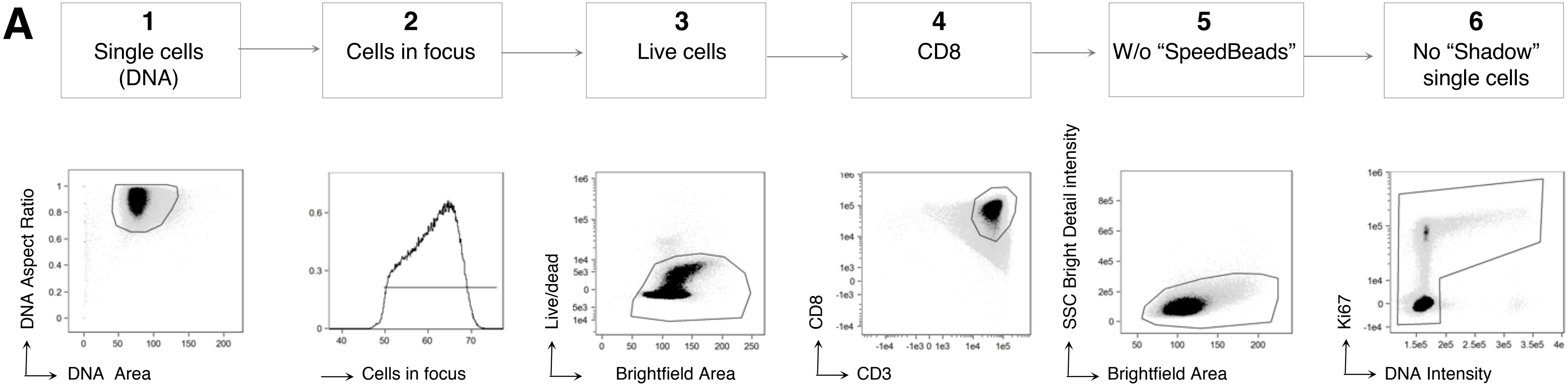

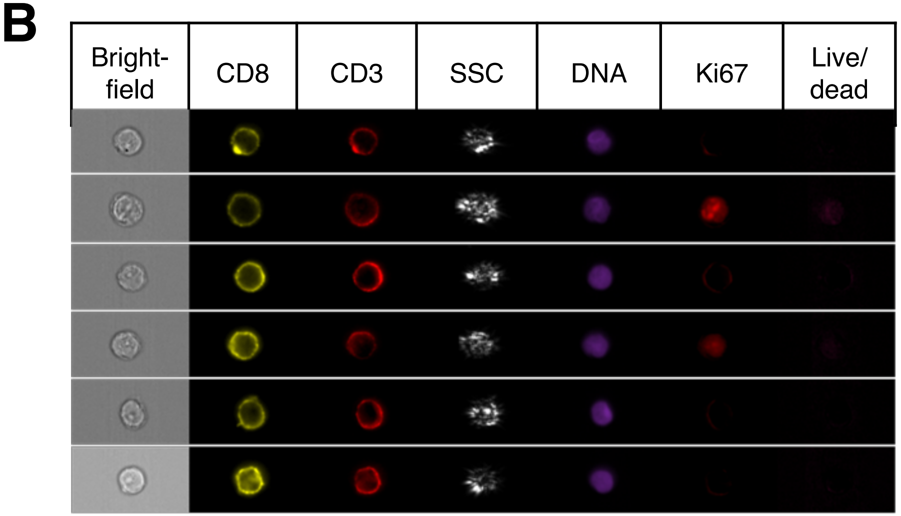

Supplemental Figure 3. Gating strategy for ImageStream analysis of human peripheral blood CD8 T cells and examples of images.

A) Scheme of the gating strategy (top), and examples (bottom) of ImageStream analysis of CD8$^+$ T cells from HD PBMC. The following cells were gated in 6 steps: single cells (Step 1); cells in focus (Step 2); live cells (Step 3); CD3$^+$CD8$^+$ cells (Step 4); cells without (w/o) “SpeedBeads” (Step 5); no “shadow” single cells (Step 6). **B)** Examples of images showing brightfield, CD8, CD3, SSC, DNA, Ki67, and live/dead staining of single CD3$^+$CD8$^+$ cells, gated as in A (Step 1 to 6).

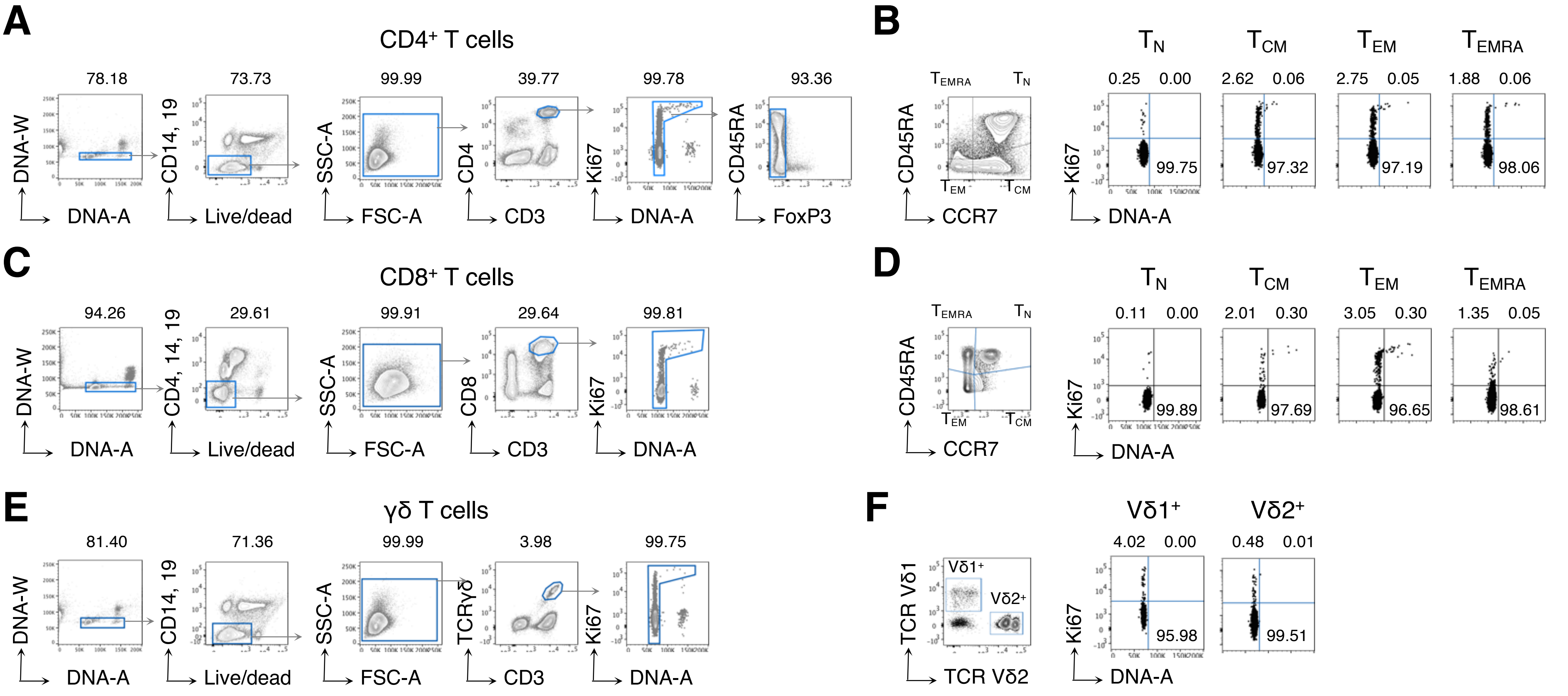

Supplemental Figure 4. Gating strategy for flow cytometry analysis of human peripheral blood T cell subsets.

A, C, E) Examples of gating strategy for flow cytometric analysis of CD4$^+$ (A); CD8$^+$ (C); and γδ T cells (E) from human PBMC in 5 steps: single cells (DNA-A/DNA-W, Step 1); live cells negative for "DUMP" markers (Step 2); lymphocytes (FSC/SSC relaxed gate, Step 3); T cell subset (Step 4); "shadow doublet" exclusion (Step 5). For conventional CD4$^+$ T cells, FoxP3$^{(-)}$ cells were gated on the CD45RA/ FoxP3 plot (Step 6, shown in A). **B, D, F)** Refined quantification of CCR7$^+$/CD45RA$^+$ (T_N), CCR7$^+$/CD45RA$^{(-)}$ (T_{CM}), CCR7$^{(-)}$/CD45RA$^{(-)}$ (T_{EM}) and CCR7$^{(-)}$/CD45RA$^+$ (T_{EMRA}) among either conventional CD4$^+$ T cells (B) or CD8$^+$ T cells (D), and of Vδ1$^+$ and Vδ2$^+$ among γδ T cells (F), with illustrative examples of cells in G_0, G_1, and S-G_2/M as assessed by Ki67/DNA profiles. Numbers represent cell percentages in the indicated gate.

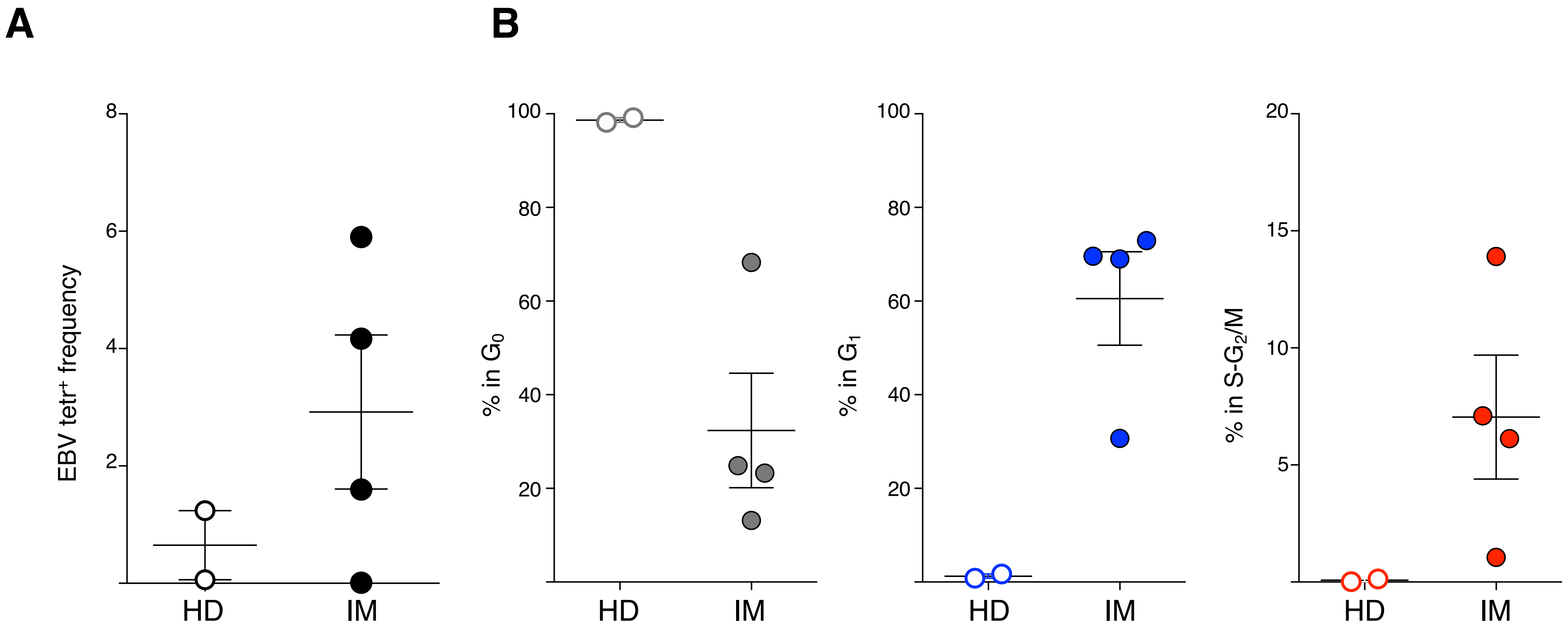

Supplemental Figure 5. Frequency of EBV tetr+ CD8 cells, and cell cycle analysis of total CD8+ T cells in IM patients.
A) Frequency of EBV tetr+ cells among total CD8+ T cells in IM patients and HD. **B)** Cell cycle analysis of total CD8+ T cells in IM patients and HD (IM patients and corresponding HD as in Fig. 4).

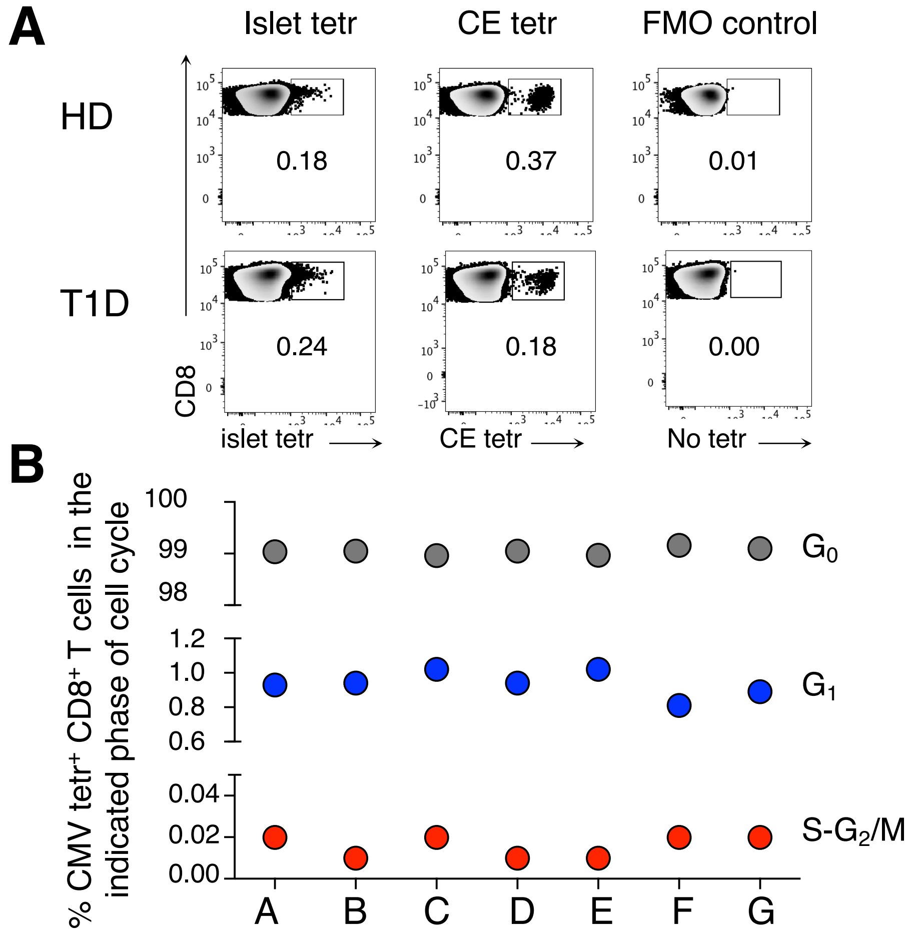

Supplemental Figure 6. Frequency of islet tetr$^+$ and CMV+EBV (CE) tetr$^+$ CD8$^+$ cells in T1D patients and HD, and T_{DS} technical reproducibility

A) Example of flow-cytometric analysis of total CD8$^+$ T cells from PBMC of an HD (top) and a T1D patient (bottom). Islet-specific CD8$^+$ T cells were identified using a pool of $PPI_{15\text{-}24}$, $InsB_{10\text{-}18}$, $GAD_{114\text{-}123}$, $IGRP_{265\text{-}273}$, and IA-$2_{797\text{-}805}$ HLA-A2 tetramers (islet tetr, left panel); CE-specific CD8$^+$ T cells using a pool of $CMV_{495\text{-}503}$ and $EBV_{280\text{-}288}$ HLA-A2 tetramers (CE tetr, centre panel). Tetr FMO control samples were analysed in parallel (right). Numbers represent cell percentages in the indicated gate. **B**) The high technical reproducibility of the T_{DS} assay. Different aliquots of identical PBMC from a CMV carrier HD were used as controls for each of 6 T1D experiments (labelled A to F) and for an IM experiment (G). The frequency of CMV-specific CD8 T cells was 0.03%, and the corresponding FMO was 0.00% in all the experiments (A to G). The panel shows the results for the percentages of CMV tetr$^+$ cells in G_0, G_1 and S-G_2/M.

Supplemental Table 1. IM patients and HD data

IDs	Sex	Diagnosis	Duration since onset of symptoms (weeks)	Duration since diagnosis (days)	Age at blood sampling (years)
I-209	F	IM	3	≤2	36
I-226	F	IM	2	≤2	18
I-258	F	IM	3	≤2	16
I-278	M	IM	2	≤2	20
I-001	F	HD	n/a	n/a	34
I-002	M	HD	n/a	n/a	26

The table shows age and sex of acute IM patients and HD included in our study. All HD were EBV+, positive for IgG against EBV Virus Capsid Antigen (VCA). All IM donors had a positive Monospot test (at Queen Elizabeth Hospital Birmingham) Duration since onset of symptoms and duration since diagnosis based on Monospot positivity are indicated for IM patients.

Supplemental Table 2. T1D patients and HD data

IDs	Sex	Diagnosis	Duration since diagnosis (days)	Age at blood sampling (years)	Definition in this study
G019	F	T1D	210	33	**T_{DS}^{hi}**
G062	F	T1D	87	22	T_{DS}^{-}
G669	F	T1D	21	30	T_{DS}^{-}
G682	F	T1D	94	19	**T_{DS}^{hi}**
G703	F	T1D	110	30	T_{DS}^{-}
G712	F	T1D	156	19	T_{DS}^{-}
G713	F	T1D	186	24	**T_{DS}^{lo}**
G716	F	T1D	72	19	**T_{DS}^{lo}**
G739	F	T1D	99	31	**T_{DS}^{lo}**
G768	F	T1D	253	30	T_{DS}^{-}
G794	F	T1D	175	25	**T_{DS}^{lo}**
G477	F	HD	n/a	25	n/a
G501	F	HD	n/a	26	n/a
G504	F	HD	n/a	32	n/a
G507	F	HD	n/a	22	n/a
G508	F	HD	n/a	32	n/a
G522	F	HD	n/a	18	n/a
G533	F	HD	n/a	25	n/a
G534	F	HD	n/a	25	n/a
G681	F	HD	n/a	29	n/a
G704	F	HD	n/a	30	n/a

The table shows age and sex of T1D patients and HD included in our study. Duration since diagnosis is indicated for T1D patients. As indicated in the right column, in this study we defined 2 main subsets of T1D patients, i.e. T_{DS}^{-} (<0.248% T_{DS}), and T_{DS}^{+} (>0.248% T_{DS}); the T_{DS}^{+} subset was subdivided in T_{DS}^{lo} (<3% T_{DS}, bold) and T_{DS}^{hi} (> 3% T_{DS}, bold underlined) (see main text for definition criteria).

Supplemental Table 3. Nanostring analysis of islet tetr^{+} CD8 T cells from PBMC

The table shows nanostring analysis of FACS-sorted islet tetr^{+} CD8 T cells from PBMC of 3 HD, 2 T1D-T_{DS}^{-}, 2 T1D- T_{DS}^{lo} (bold), and 2 T1D- T_{DS}^{hi} (bold underlined) donors using commercially available CAR-T gene panel (see main text for T1D patient definition criteria).

	G501<br>HD	G681<br>HD	G704<br>HD	G703<br>T_{DS}^{-}	G669<br>T_{DS}^{-}	G716<br>**T_{DS}^{lo}**	G713<br>**T_{DS}^{lo}**	G019<br>**<u>T_{DS}^{hi}</u>**	G682<br>**<u>T_{DS}^{hi}</u>**
Probe Name									
DUSP1	1428.22	1875.53	2487.66	1697.62	1573.47	1678.84	1719.21	2000.4	1647.63
FOS	1295.86	1149.34	2209.86	1492.43	1475.21	1507.78	1624.67	1422.75	1219.18
RPL23	890.44	835.06	1332.95	1094.33	1001.85	1142.85	1132.72	782.38	905.74
TRBC1/2	790.75	665.58	943.86	968.06	874.82	807.25	870.11	959.53	765.3
FOSB	713.69	912.51	1492.76	811.98	1124.08	753.48	976.9	984.35	937.28
PTPRC	698.61	445.59	768.97	417.39	686.67	680.17	542.72	800.99	658.44
RPL3	546.99	450.08	738.01	599.78	719.03	582.42	656.52	476.32	582.11
CXCR4	485.01	1124.64	1424.15	1434.56	2529.78	1831.17	1750.72	1016.74	1595.72
PPIA	485.01	410.8	460.21	441.94	460.18	482.23	511.21	696.9	602.47
HLA-DRA	454.85	322.13	213.37	131.53	83.89	216.68	239.85	107.53	198.45
NKG7	453.18	451.2	213.37	250.78	310.38	313.61	224.09	1100.84	730.7
NFKBIA	443.12	1043.83	620.87	448.96	905.98	470.83	621.51	619.7	531.23
TRAC	419.67	153.77	466.07	477.02	237.28	400.77	395.66	547.32	429.46
UBB	412.13	413.04	424.23	431.42	511.71	472.45	460.44	547.32	524.11
HLA-E	407.1	428.76	539.7	427.91	430.22	517.26	477.95	457.71	420.3
CXCL8	386.16	937.2	25.94	66.64	168.97	109.97	108.54	122.01	141.46
JUNB	353.49	595.99	1238.39	1029.45	980.28	937.58	1045.18	509.41	531.23
PRF1	323.34	266.01	87.02	124.52	95.87	114.04	75.28	567.31	330.75
CD8A	318.31	264.89	405.82	426.16	621.96	424.39	329.14	596.26	494.59
ITGB2	304.91	127.95	206.68	168.36	129.43	181.65	159.32	246.09	192.34
IL32	293.18	215.5	424.23	445.45	353.52	395.88	483.2	726.54	453.89
GNAI2	274.75	177.34	251.86	175.37	139.01	207.72	222.34	242.64	214.73
HLA-DRB1	273.08	151.52	102.08	71.9	58.72	109.15	103.29	94.44	108.89
KLRK1	273.08	98.77	181.58	191.16	141.41	190.61	166.32	214.38	188.27
TYROBP	264.7	206.52	193.29	33.32	27.56	87.97	56.02	51.01	76.33
CCL5	261.35	202.03	230.94	294.63	255.26	243.56	239.85	564.55	501.72
LTB	218.63	99.89	283.66	366.53	188.15	196.31	304.63	152.34	129.25
OAZ1	213.6	130.2	179.06	171.87	146.2	167.8	150.56	230.23	199.47
SELL	210.25	84.18	206.68	192.91	162.98	179.21	147.06	198.52	173.01
NME2	203.55	189.69	186.6	242.02	183.35	180.84	208.34	197.83	204.55
JUN	198.53	312.03	443.48	212.2	359.51	166.99	269.61	563.17	324.64
ALDOA	196.01	166.11	175.72	145.56	148.6	144.99	222.34	184.05	188.27
CD3E	193.5	173.97	230.94	261.31	165.38	217.49	211.84	268.14	242.21
CCL3/L1	192.66	243.56	13.39	19.29	52.73	46.43	43.77	164.75	64.11

CD3D	184.29	144.79	216.72	217.46	161.78	188.98	215.34	301.23	213.71
CD45RA	184.29	203.15	231.78	138.55	123.43	197.13	155.81	137.17	121.1
FCGR3A/B	180.94	209.89	241.82	29.81	27.56	86.35	24.51	89.61	37.65
SLC25A6	169.21	191.93	217.56	187.65	168.97	181.65	239.85	181.29	203.54
GZMA	167.53	117.85	95.39	77.16	73.1	79.01	94.54	516.99	321.59
IL10RA	165.86	109.99	184.92	133.28	117.44	160.47	87.54	181.98	132.3
KLRG1	164.18	92.04	144.76	124.52	76.7	83.9	66.53	224.72	86.5
RAC2	162.51	99.89	166.51	149.07	87.48	135.22	113.8	230.92	182.17
CDC42	160.83	141.42	176.55	122.76	117.44	168.62	164.57	173.71	188.27
PGK1	159.99	176.22	201.66	199.93	279.22	254.15	206.59	175.78	186.24
JAK1	151.62	162.75	207.51	159.59	212.11	214.23	192.58	184.05	166.9
CD69	145.75	300.8	289.52	306.9	583.61	216.68	332.64	441.85	410.13
CASP8	142.4	147.03	110.45	99.96	104.26	141.74	127.8	161.99	152.65
ZAP70	142.4	111.12	184.92	161.34	171.37	170.25	150.56	182.67	144.51
IL16	140.73	51.63	133.04	121.01	95.87	130.33	98.04	117.87	78.36
TGFB1	140.73	207.64	229.27	201.68	527.29	263.92	206.59	218.51	245.26
ADAR	138.21	131.32	142.25	140.3	121.04	147.44	143.56	141.31	131.28
CTNNB1	138.21	151.52	129.7	89.44	99.47	131.15	126.05	110.29	115
IRF1	135.7	173.97	215.05	112.24	341.54	158.84	155.81	173.02	191.32
SKP1	135.7	138.05	196.64	128.02	188.15	162.1	150.56	144.07	139.42
CD7	134.86	78.57	147.27	215.71	168.97	224.82	166.32	142	129.25
IL2RG	130.68	101.02	148.11	156.08	101.86	139.29	162.82	245.4	186.24
LAMP1	129.84	127.95	127.19	99.96	143.81	114.86	120.8	122.7	122.12
IFI30	129	69.59	102.92	19.29	5.99	49.69	29.76	14.48	22.39
LEF1	120.62	85.3	184.92	149.07	113.85	204.46	173.32	61.35	88.54
SERINC1	119.79	169.48	138.06	108.73	200.13	166.17	169.82	113.74	126.19
COX6C	118.11	122.34	133.88	164.85	130.62	122.19	152.31	161.99	136.37
CTSW	114.76	43.77	127.19	187.65	56.32	194.68	199.58	270.21	141.46
ACADVL	113.08	77.45	92.04	91.19	86.28	91.23	73.53	102.02	93.63
PECAM1	112.25	78.57	157.31	59.63	35.95	102.64	45.52	57.21	63.1
CCL4/L1	108.06	129.08	56.9	29.81	81.49	74.13	50.77	166.13	106.86
LCK	108.06	47.14	135.55	112.24	77.89	94.49	99.79	131.66	107.87
GRK2	104.71	65.1	112.96	89.44	89.88	83.09	70.03	97.19	89.56
TKT	103.87	65.1	87.86	29.81	26.36	53.76	59.52	44.81	62.08
TRGC2	103.03	26.94	42.67	40.34	17.98	8.15	64.78	124.08	68.18
CYBB	100.52	69.59	60.25	12.28	10.79	35.84	28.01	11.03	12.21
MAX	100.52	90.91	117.98	101.72	103.06	116.48	106.79	108.91	100.75
IL7R	99.68	42.65	102.92	103.47	133.02	104.27	134.81	103.4	85.49
RUNX3	98.84	101.02	97.06	126.27	225.3	169.43	129.55	115.81	132.3
CD14	98.01	21.33	10.04	31.57	8.39	12.22	35.01	15.16	25.44
CD8B	98.01	81.94	153.96	84.18	105.46	131.96	134.81	150.96	142.48
PLCG1	98.01	62.85	136.39	98.21	69.51	102.64	126.05	99.26	86.5
DOCK2	96.33	77.45	125.51	63.13	80.29	85.53	84.03	95.13	94.64
ID2	96.33	106.63	136.39	115.75	119.84	117.3	131.3	130.97	116.02

FYN	92.98	75.2	116.31	106.98	216.91	140.92	98.04	124.77	111.94
PTPN6	92.98	59.49	115.47	57.87	15.58	57.83	42.02	62.04	54.95
PTK2B	92.14	84.18	87.02	77.16	52.73	79.01	82.28	97.19	84.47
SIK1	92.14	308.66	369.84	313.92	435.01	388.55	350.14	124.77	173.01
POLR2A	92.14	83.06	132.21	103.47	131.82	106.71	133.05	80.65	107.87
COX5B	91.31	76.32	70.29	91.19	58.72	92.05	87.54	74.45	87.52
GZMK	90.47	41.53	150.62	115.75	162.98	80.64	127.8	267.46	144.51
NDUFA4	90.47	76.32	76.14	80.67	53.93	85.53	84.03	100.64	68.18
GZMB	89.63	189.69	19.25	21.04	40.74	26.07	33.26	226.79	143.49
COX7C	87.95	89.79	92.88	94.7	105.46	86.35	105.04	97.19	83.45
MIF	87.95	96.53	100.41	115.75	103.06	84.72	113.8	126.83	87.52
IL6ST	87.12	94.28	141.41	64.89	103.06	96.93	66.53	43.43	63.1
TCF7	87.12	55	143.08	129.78	76.7	149.07	110.3	65.49	81.41
CD3G	86.28	77.45	78.65	94.7	77.89	87.16	63.03	129.59	148.58
CD45R0	85.44	8.98	66.94	94.7	128.23	62.72	75.28	188.87	158.76
STAT1	85.44	75.2	143.08	56.12	67.11	105.89	82.28	97.88	93.63
CX3CR1	84.6	28.06	47.69	8.77	7.19	10.59	5.25	66.86	40.71
GADD45B	84.6	126.83	166.51	96.46	313.98	128.7	155.81	88.23	134.33
TRIM22	84.6	98.77	93.72	50.86	69.51	66.8	92.79	104.09	61.06
GLS	82.93	74.08	94.55	66.64	85.09	104.27	91.04	109.6	78.36
HSPE1	82.93	70.71	117.15	85.93	117.44	126.26	122.55	97.19	88.54
STAT6	82.93	59.49	84.51	56.12	56.32	72.5	61.28	74.45	80.4
CTSD	81.25	53.88	69.45	33.32	40.74	47.25	71.78	58.59	59.03
LDHA	80.42	86.42	74.47	92.95	188.15	121.37	119.05	117.87	124.16
UBE2V1	80.42	87.55	98.74	94.7	81.49	79.83	98.04	118.56	96.68
COX6B1	79.58	48.26	79.49	57.87	50.33	76.57	85.79	90.99	73.27
COX4I1	77.06	107.75	120.49	77.16	88.68	120.56	145.31	95.13	95.66
CD6	76.23	92.04	76.14	57.87	141.41	113.23	75.28	56.52	39.69
PSMB10	76.23	53.88	83.68	50.86	41.94	60.28	63.03	122.7	61.06
COX7B	75.39	65.1	62.76	56.12	61.12	65.17	85.79	90.3	79.38
NDUFA1	75.39	67.34	80.33	82.43	52.73	85.53	70.03	104.78	106.86
IFITM3	74.55	144.79	23.43	24.55	22.77	64.35	45.52	15.85	25.44
PGAM1	72.88	67.34	82.84	63.13	53.93	62.72	71.78	108.22	93.63
PKM	72.88	69.59	79.49	70.15	57.52	76.57	105.04	74.45	88.54
GZMH	72.04	84.18	19.25	19.29	26.36	39.91	19.26	142	128.23
IRF9	72.04	88.67	67.78	33.32	56.32	69.24	61.28	48.25	51.9
SERINC3	72.04	80.81	63.59	64.89	82.69	82.27	68.28	78.58	66.15
IL2RB	71.2	26.94	101.25	166.61	127.03	128.7	71.78	116.49	81.41
CD68	69.53	56.12	54.39	17.54	14.38	23.62	31.51	13.79	17.3
TYK2	69.53	55	44.35	47.35	46.74	33.4	38.52	42.05	29.51
WAS	68.69	61.73	92.88	45.6	41.94	50.5	66.53	86.16	55.97
CHMP4A	67.85	39.28	66.1	49.1	61.12	72.5	56.02	69.62	59.03
ERAP2	67.01	48.26	37.65	52.61	34.75	96.93	106.79	133.73	66.15
GATA3	67.01	60.61	96.23	129.78	128.23	89.6	115.55	90.99	90.57

SP100	67.01	46.02	68.61	36.83	34.75	51.32	59.52	57.21	60.04
ADD1	66.18	49.39	72.8	56.12	34.75	59.46	71.78	73.76	72.26
GPI	65.34	40.41	73.63	50.86	45.54	53.76	49.02	75.82	65.13
PTGER4	64.5	138.05	176.55	184.14	260.05	185.72	203.08	98.57	118.05
SDHA	64.5	53.88	68.61	61.38	59.92	71.68	70.03	79.96	58.01
HIF1A	62.82	150.4	103.76	101.72	152.19	161.29	131.3	88.92	85.49
TBX21	62.82	31.43	39.33	49.1	39.55	52.13	45.52	84.79	61.06
NFKB2	59.47	102.14	66.94	54.37	185.75	70.05	80.53	43.43	55.97
SERPINB9	58.64	52.75	76.98	33.32	57.52	40.73	19.26	70.31	67.17
IKZF2	56.96	16.84	32.63	85.93	13.18	43.17	43.77	35.84	36.64
PSMA2	56.96	46.02	36.82	43.84	35.95	52.13	78.78	86.85	71.24
PYCR2	56.96	22.45	57.74	49.1	34.75	41.54	47.27	54.46	54.95
COX7A2	56.12	49.39	66.1	75.41	69.51	96.93	115.55	79.27	91.59
KLRB1	56.12	84.18	52.72	66.64	76.7	39.91	57.77	260.56	100.75
PLCB2	56.12	38.16	75.31	36.83	35.95	43.17	29.76	33.78	52.92
STAT4	56.12	75.2	55.23	42.09	124.63	71.68	35.01	62.73	54.95
TRIM33	54.45	46.02	56.9	42.09	65.91	62.72	50.77	62.04	43.76
AHR	52.77	33.67	46.86	33.32	39.55	26.88	35.01	23.44	28.5
CSF3R	52.77	26.94	15.9	14.03	2.4	9.77	7	10.34	12.21
ITGAM	52.77	28.06	20.92	49.1	15.58	36.66	21.01	22.06	32.57
PRKCB	52.77	35.92	62.76	29.81	50.33	56.21	57.77	78.58	67.17
SH3BP2	52.77	39.28	44.35	19.29	10.79	36.66	28.01	33.09	19.34
USP15	52.77	59.49	41	61.38	53.93	71.68	50.77	50.32	43.76
IFNGR1	51.94	79.69	61.08	47.35	68.31	61.09	56.02	57.9	61.06
NEDD8	51.94	47.14	61.92	47.35	31.16	57.02	57.77	67.55	65.13
SLAMF6	51.94	25.82	28.45	31.57	21.57	23.62	40.27	84.79	55.97
TGFBR2	51.94	40.41	71.96	61.38	61.12	66.8	56.02	42.74	42.74
CD96	51.1	39.28	57.74	49.1	68.31	61.09	49.02	60.66	72.26
DECR1	50.26	20.2	46.86	38.58	23.97	33.4	31.51	62.04	35.62
FOXO1	50.26	84.18	97.9	56.12	87.48	82.27	85.79	54.46	67.17
PML	50.26	32.55	41	36.83	17.98	34.21	28.01	34.47	37.65
PTGER2	50.26	24.69	54.39	56.12	13.18	19.55	28.01	65.49	28.5
STAT3	50.26	50.51	51.04	38.58	75.5	62.72	47.27	57.9	46.81
ATP6V1F	49.42	47.14	53.55	31.57	43.14	32.58	66.53	59.97	36.64
CALM1	49.42	50.51	63.59	54.37	86.28	72.5	80.53	73.07	60.04
CD99	49.42	50.51	50.21	31.57	49.13	64.35	54.27	71.69	82.43
MAP2K2	49.42	34.79	43.51	36.83	43.14	51.32	36.77	42.74	46.81
TRAV14	49.42	10.1	35.98	26.31	20.37	25.25	26.26	32.4	60.04
GLUD1/2	48.58	40.41	61.08	35.07	35.95	48.87	40.27	55.15	54.95
NR3C1	48.58	34.79	47.69	36.83	65.91	59.46	43.77	40.67	41.72
TRAV19	48.58	24.69	30.12	54.37	25.17	35.84	29.76	52.39	27.48
HLA-DQA1	47.75	1.12	46.02	1.75	2.4	35.84	1.75	2.76	39.69
LILRA5	47.75	50.51	36.82	8.77	3.6	21.99	10.5	3.45	9.16
PSMA6	47.75	46.02	40.16	40.34	34.75	32.58	42.02	58.59	50.88

MDH2	46.91	35.92	42.67	45.6	33.55	34.21	54.27	52.39	38.67
AKT2	46.07	59.49	57.74	64.89	55.13	85.53	66.53	55.83	51.9
LAMP2	46.07	39.28	31.8	42.09	29.96	43.99	43.77	42.05	50.88
TGFBR1	46.07	28.06	35.98	42.09	21.57	28.51	45.52	53.77	35.62
BCL2	44.4	44.9	48.53	52.61	35.95	51.32	36.77	33.09	30.53
IL1B	44.4	331.11	12.55	35.07	62.32	33.4	42.02	36.53	42.74
PFKL	44.4	46.02	55.23	49.1	43.14	60.28	59.52	41.36	61.06
CD84	43.56	21.33	51.04	50.86	34.75	37.47	43.77	51.01	49.87
NDUFB9	43.56	28.06	43.51	36.83	34.75	38.29	50.77	58.59	63.1
PIK3R1	43.56	47.14	74.47	149.07	167.77	209.35	106.79	53.08	66.15
RORA	43.56	59.49	55.23	31.57	92.28	49.69	36.77	97.19	76.33
TRAT1	43.56	37.04	49.37	36.83	52.73	39.1	35.01	37.91	39.69
TRBV28	43.56	286.21	65.27	14.03	1.2	8.96	15.76	8.27	10.18
AKT1	42.72	23.57	32.63	28.06	29.96	31.77	36.77	42.74	42.74
CREB1	42.72	26.94	31.8	29.81	32.36	27.7	15.76	40.67	36.64
IFNAR1	42.72	15.71	38.49	22.8	23.97	26.88	28.01	24.13	33.58
RELA	42.72	52.75	41.84	47.35	89.88	48.87	47.27	51.01	39.69
TP53	42.72	16.84	35.14	22.8	22.77	30.95	36.77	34.47	33.58
TRDC	42.72	3.37	15.06	19.29	14.38	8.15	5.25	97.88	46.81
DAP3	41.05	44.9	40.16	40.34	45.54	40.73	43.77	39.29	42.74
MAML2	41.05	47.14	81.16	68.4	53.93	73.31	54.27	25.5	32.57
NOTCH2	41.05	20.2	41	12.28	21.57	26.07	24.51	31.71	18.32
RBX1	41.05	40.41	57.74	33.32	55.13	38.29	40.27	48.94	55.97
IRF7	40.21	56.12	25.1	33.32	56.32	30.14	40.27	36.53	20.35
HSPA9	39.37	31.43	40.16	45.6	52.73	41.54	40.27	32.4	27.48
ACTN1	38.53	44.9	42.67	54.37	37.15	54.58	42.02	21.37	27.48
NDUFAB1	38.53	32.55	37.65	21.04	28.76	40.73	52.52	51.01	40.71
OAS2	38.53	19.08	30.12	21.04	11.98	15.48	12.26	39.98	27.48
TIMP1	38.53	32.55	37.65	26.31	13.18	31.77	17.51	15.16	22.39
ATP5PD	37.69	47.14	40.16	22.8	40.74	35.84	47.27	44.81	41.72
DDIT4	37.69	86.42	82.84	85.93	194.14	95.31	108.54	57.9	70.22
SMAD2	37.69	25.82	45.18	26.31	23.97	30.14	42.02	42.74	28.5
ATG7	36.86	26.94	27.61	35.07	10.79	30.95	21.01	36.53	28.5
VAV1	36.86	23.57	45.18	28.06	27.56	31.77	24.51	33.78	40.71
EGR1	36.02	43.77	56.9	21.04	31.16	22.81	29.76	76.51	28.5
LAIR1	36.02	13.47	27.61	22.8	8.39	16.29	22.76	30.33	28.5
TFRC	36.02	62.85	62.76	77.16	113.85	60.28	57.77	48.25	56.99
TRBV20-1	36.02	35.92	46.86	33.32	38.35	40.73	26.26	49.63	38.67
CS	35.18	37.04	35.98	43.84	31.16	43.99	36.77	55.83	54.95
GZMM	35.18	19.08	40.16	31.57	50.33	32.58	24.51	35.84	34.6
LILRB2	35.18	66.22	45.18	1.75	3.6	27.7	7	5.51	6.11
STAT5B	35.18	23.57	45.18	38.58	50.33	39.91	49.02	35.16	52.92
STK11	35.18	30.3	35.14	28.06	20.37	24.44	49.02	34.47	27.48
TRAV8-3	35.18	10.1	25.1	33.32	4.79	30.95	12.26	21.37	22.39

UBE2I	35.18	21.33	54.39	49.1	50.33	43.99	56.02	38.6	52.92
CCR7	34.34	53.88	66.94	47.35	105.46	57.02	61.28	18.61	28.5
MAP3K7	34.34	15.71	30.12	26.31	27.56	33.4	28.01	35.84	29.51
PARP1	34.34	17.96	21.76	26.31	19.17	26.07	35.01	37.91	29.51
TRGV3/5	34.34	14.59	31.8	64.89	20.37	49.69	12.26	56.52	27.48
UQCRQ	34.34	25.82	34.31	36.83	25.17	30.14	47.27	37.91	33.58
IKBKE	33.51	17.96	32.63	35.07	23.97	23.62	28.01	28.26	35.62
TPR	33.51	21.33	29.29	29.81	17.98	25.25	28.01	24.13	26.46
TRAV8-2	33.51	15.71	46.86	24.55	11.98	46.43	31.51	38.6	29.51
ABCF1	33.51	57.24	51.04	56.12	82.69	48.06	70.03	50.32	45.8
ACSL5	32.67	17.96	28.45	24.55	17.98	24.44	29.76	37.22	39.69
MPC2	32.67	31.43	33.47	40.34	17.98	35.84	50.77	37.22	32.57
TNF	32.67	89.79	23.43	17.54	49.13	18.74	17.51	54.46	28.5
TREM1	32.67	12.35	11.71	7.01	8.39	7.33	7	2.76	9.16
XAF1	32.67	30.3	21.76	10.52	9.59	10.59	21.01	19.3	18.32
CD247	31.83	48.26	42.67	31.57	45.54	48.87	45.52	64.11	48.85
IL4R	31.83	22.45	19.25	42.09	95.87	78.2	38.52	22.06	30.53
LAT	31.83	13.47	40.16	14.03	16.78	35.03	31.51	44.12	21.37
PIK3C3	31.83	30.3	38.49	33.32	29.96	35.84	12.26	32.4	27.48
SLC3A2	31.83	56.12	43.51	52.61	56.32	47.25	49.02	37.22	39.69
TRAV12-2	31.83	6.73	32.63	36.83	22.77	59.46	42.02	61.35	32.57
IFNGR2	30.99	31.43	30.96	17.54	8.39	13.85	12.26	9.65	7.12
MAPK3	30.99	12.35	25.94	15.78	13.18	19.55	22.76	19.3	16.28
PSMA3	30.99	31.43	30.96	28.06	35.95	38.29	31.51	48.94	48.85
TET2	30.99	43.77	29.29	24.55	40.74	42.36	26.26	19.99	17.3
TRAV17	30.99	8.98	16.74	21.04	20.37	22.81	7	33.78	25.44
CXCL2	30.16	61.73	4.18	10.52	21.57	30.95	28.01	7.58	28.5
DHRS4	30.16	12.35	14.22	15.78	16.78	20.36	24.51	31.02	21.37
IL21R	30.16	19.08	20.92	42.09	172.57	78.2	33.26	18.61	23.41
MYC	30.16	11.22	51.88	29.81	27.56	15.48	24.51	26.88	23.41
NMT1	30.16	25.82	25.94	19.29	22.77	29.32	26.26	39.29	32.57
SCD	30.16	28.06	20.92	17.54	23.97	15.48	31.51	19.3	18.32
VSIR	30.16	23.57	37.65	21.04	10.79	34.21	12.26	17.92	22.39
DGLUCY	29.32	19.08	40.16	17.54	26.36	25.25	28.01	39.29	23.41
DIABLO	29.32	30.3	34.31	24.55	37.15	33.4	22.76	28.26	30.53
FNIP1	29.32	35.92	35.14	24.55	45.54	39.91	36.77	17.23	36.64
HADHB	29.32	19.08	30.12	26.31	28.76	24.44	26.26	33.78	26.46
NDUFA6	29.32	40.41	51.04	33.32	40.74	39.91	45.52	51.7	33.58
SDHB	29.32	30.3	53.55	33.32	43.14	53.76	50.77	37.22	39.69
SOS2	29.32	16.84	30.12	26.31	53.93	35.03	33.26	32.4	28.5
TRBV4-3	29.32	4.49	28.45	35.07	37.15	1	24.51	35.16	40.71
UBE2F	29.32	22.45	19.25	19.29	26.36	34.21	19.26	25.5	23.41
ALDH3A2	28.48	19.08	31.8	33.32	16.78	31.77	26.26	24.82	25.44
IL12RB1	28.48	13.47	17.57	7.01	22.77	11.4	28.01	33.09	27.48

MAGED1	28.48	14.59	26.78	17.54	27.56	25.25	35.01	26.88	22.39
SMARCA4	28.48	24.69	20.92	43.84	23.97	32.58	21.01	31.02	23.41
TFAM	28.48	28.06	22.59	19.29	17.98	26.07	36.77	27.57	16.28
TRGV2	28.48	24.69	16.74	26.31	9.59	17.92	29.76	28.26	44.78
CDC26	27.64	13.47	19.25	15.78	10.79	21.18	26.26	22.75	27.48
MT2A	27.64	48.26	49.37	47.35	49.13	38.29	22.76	37.22	34.6
S100A12	27.64	6.73	1.67	5.26	1.2	1.63	3.5	4.83	8.14
SH2D1A	27.64	25.82	35.98	38.58	46.74	21.99	31.51	59.97	49.87
GARS	26.81	30.3	20.92	12.28	28.76	28.51	42.02	27.57	32.57
MX1	26.81	38.16	41	14.03	23.97	35.84	15.76	39.29	33.58
SEC22B	26.81	25.82	20.08	8.77	29.96	17.92	28.01	17.92	19.34
TRBV7-9	26.81	10.1	26.78	33.32	21.57	25.25	15.76	26.88	34.6
TRIM25	26.81	25.82	17.57	12.28	15.58	20.36	17.51	26.19	13.23
CDKN1A	25.97	76.32	35.14	21.04	85.09	36.66	56.02	22.75	26.46
HACD4	25.97	16.84	33.47	24.55	15.58	22.81	31.51	37.22	27.48
ISG15	25.97	37.04	20.08	12.28	19.17	13.03	15.76	23.44	20.35
RAE1	25.97	28.06	19.25	14.03	34.75	30.95	22.76	29.64	24.42
TLR2	25.97	15.71	10.04	3.51	7.19	11.4	15.76	2.76	4.07
TRBV19	25.97	8.98	22.59	19.29	46.74	42.36	35.01	22.06	21.37
NCAPD2	25.13	17.96	22.59	14.03	23.97	16.29	49.02	47.56	28.5
TRAF5	25.13	10.1	20.08	33.32	14.38	30.14	31.51	24.13	21.37
TRGC1	25.13	12.35	15.06	22.8	25.17	46.43	22.76	23.44	19.34
FASN	24.29	28.06	25.94	47.35	91.08	39.91	38.52	19.3	23.41
NOTCH1	24.29	19.08	18.41	7.01	10.79	20.36	33.26	18.61	25.44
OAS1	24.29	21.33	20.08	7.01	3.6	13.03	8.75	15.85	10.18
OAT	24.29	21.33	37.65	26.31	37.15	33.4	33.26	20.68	28.5
PDK1	24.29	47.14	40.16	28.06	25.17	43.99	35.01	28.26	22.39
CHMP3	23.45	28.06	39.33	33.32	26.36	24.44	35.01	33.09	38.67
KLRC1/2	23.45	10.1	16.74	21.04	20.37	23.62	21.01	11.72	14.25
MAF	23.45	28.06	28.45	21.04	21.57	22.81	21.01	47.56	35.62
MAP2K7	23.45	29.18	30.96	35.07	46.74	33.4	35.01	24.82	31.55
MTHFD1	23.45	20.2	27.61	26.31	15.58	23.62	45.52	33.78	21.37
OPA1	23.45	29.18	41	28.06	14.38	36.66	29.76	19.99	34.6
PRKCD	23.45	19.08	18.41	14.03	10.79	8.15	24.51	14.48	13.23
STAT5A	23.45	20.2	30.96	29.81	47.94	35.03	21.01	25.5	34.6
TRGV8	23.45	29.18	7.53	19.29	5.99	15.48	15.76	14.48	2.04
TRIM26	23.45	32.55	25.94	24.55	21.57	29.32	28.01	29.64	29.51
UQCR10	23.45	8.98	20.08	17.54	16.78	21.18	19.26	17.92	24.42
IRF3	22.62	23.57	31.8	26.31	21.57	17.11	28.01	26.88	14.25
LILRB3	22.62	14.59	14.22	8.77	10.79	7.33	15.76	4.14	9.16
TIGIT	22.62	7.86	21.76	17.54	19.17	18.74	24.51	26.88	24.42
TRAF6	22.62	28.06	20.92	12.28	8.39	22.81	12.26	18.61	17.3
TSC2	22.62	20.2	19.25	22.8	19.17	19.55	19.26	19.99	16.28
ATP5MG	21.78	23.57	18.41	22.8	21.57	22.81	38.52	25.5	20.35

CCL20	21.78	31.43	1	3.51	10.79	7.33	3.5	21.37	6.11
CCNC	21.78	28.06	20.08	35.07	22.77	26.07	36.77	29.64	28.5
GNLY	21.78	19.08	5.02	7.01	17.98	24.44	3.5	4.83	5.09
ICAM1	21.78	52.75	8.37	10.52	27.56	15.48	22.76	10.34	15.27
MTHFS	21.78	13.47	24.27	12.28	17.98	8.15	15.76	13.1	17.3
TGIF2	21.78	23.57	20.08	26.31	40.74	23.62	19.26	8.96	13.23
TOLLIP	21.78	11.22	18.41	15.78	19.17	15.48	12.26	15.85	8.14
TRBV29-1	21.78	13.47	53.55	26.31	23.97	44.8	29.76	17.92	28.5
CD4	20.94	7.86	12.55	5.26	3.6	6.52	7	4.14	6.11
CSF2RA	20.94	14.59	10.88	1.75	2.4	8.15	8.75	2.76	4.07
GFM1	20.94	20.2	23.43	8.77	13.18	30.95	21.01	26.19	21.37
LAMTOR1	20.94	19.08	22.59	10.52	9.59	12.22	17.51	15.16	14.25
LILRA1	20.94	12.35	32.63	3.51	1.2	12.22	5.25	3.45	4.07
MID1IP1	20.94	21.33	11.71	10.52	7.19	13.85	15.76	15.85	13.23
NAA20	20.94	22.45	13.39	10.52	21.57	11.4	14.01	28.95	23.41
ALDOC	20.1	11.22	19.25	28.06	19.17	25.25	19.26	11.03	10.18
CTNND1	20.1	15.71	19.25	10.52	11.98	14.66	8.75	5.51	12.21
DVL2	20.1	7.86	12.55	5.26	4.79	9.77	15.76	11.03	12.21
IFNG	20.1	22.45	19.25	15.78	23.97	13.85	7	55.83	65.13
NFATC2IP	20.1	26.94	29.29	24.55	22.77	30.14	28.01	26.88	26.46
PCK2	20.1	21.33	16.74	8.77	9.59	2.44	8.75	12.41	8.14
TRAV12-3	20.1	7.86	22.59	14.03	5.99	17.92	14.01	12.41	8.14
TRBV27	20.1	16.84	29.29	29.81	5.99	9.77	19.26	14.48	27.48
TRBV4-1	20.1	10.1	5.02	21.04	2.4	21.99	17.51	15.16	17.3
CTNNA1	19.27	19.08	14.22	15.78	11.98	15.48	17.51	13.79	27.48
PFKP	19.27	16.84	19.25	28.06	14.38	19.55	14.01	18.61	28.5
TRAV1-2	19.27	5.61	12.55	19.29	5.99	11.4	17.51	47.56	11.19
UBA5	19.27	17.96	22.59	10.52	16.78	24.44	31.51	25.5	19.34
BCL6	18.43	21.33	17.57	15.78	16.78	22.81	22.76	11.72	12.21
BID	18.43	15.71	19.25	8.77	7.19	7.33	7	6.2	5.09
CD27	18.43	4.49	16.74	21.04	7.19	14.66	12.26	19.99	20.35
GART	18.43	11.22	18.41	14.03	11.98	14.66	19.26	18.61	4.07
JAK2	18.43	15.71	10.04	8.77	9.59	11.4	10.5	15.85	14.25
NCR3	18.43	20.2	17.57	26.31	16.78	27.7	42.02	26.19	13.23
NFATC1	18.43	14.59	17.57	12.28	19.17	27.7	12.26	14.48	9.16
NME1	18.43	25.82	18.41	26.31	14.38	17.92	22.76	15.16	24.42
NPRL3	18.43	20.2	30.96	28.06	13.18	21.18	31.51	24.13	20.35
NT5E	18.43	16.84	26.78	26.31	13.18	24.44	29.76	11.03	18.32
SMAD3	18.43	23.57	36.82	21.04	33.55	13.85	40.27	55.15	36.64
TIMM17A	18.43	24.69	21.76	26.31	27.56	31.77	21.01	17.23	21.37
TRBV9	18.43	5.61	25.94	21.04	14.38	19.55	17.51	24.13	15.27
TBP	18.43	10.1	14.22	14.03	10.79	24.44	7	15.85	15.27
ADORA2A	17.59	15.71	22.59	10.52	11.98	19.55	12.26	15.85	14.25
CXCR3	17.59	14.59	22.59	33.32	56.32	35.03	38.52	46.18	33.58

LPAR6	17.59	11.22	10.88	12.28	5.99	12.22	5.25	13.79	22.39
PRDM1	17.59	32.55	13.39	10.52	39.55	14.66	19.26	55.83	32.57
SLAMF7	17.59	15.71	25.1	12.28	16.78	13.85	19.26	52.39	23.41
SLC27A3	17.59	8.98	12.55	19.29	2.4	13.03	10.5	11.72	2.04
SMURF1	17.59	14.59	12.55	7.01	26.36	16.29	12.26	9.65	11.19
SOCS4	17.59	13.47	8.37	19.29	19.17	17.11	17.51	14.48	13.23
STAT2	17.59	12.35	16.74	5.26	7.19	4.89	10.5	9.65	10.18
TIMM23	17.59	37.04	20.08	24.55	25.17	22.81	21.01	22.06	20.35
TRAV41	17.59	4.49	15.06	29.81	7.19	10.59	12.26	2.76	6.11
G6PD	17.59	20.2	28.45	29.81	19.17	16.29	29.76	19.99	22.39
NRDE2	17.59	15.71	18.41	12.28	19.17	17.92	19.26	13.1	17.3
ATG14	16.75	31.43	20.08	26.31	23.97	30.14	17.51	17.92	23.41
CD300E	16.75	34.79	21.76	1.75	3.6	9.77	3.5	1.38	4.07
FH	16.75	23.57	14.22	21.04	17.98	16.29	24.51	22.06	27.48
FLCN	16.75	5.61	15.06	12.28	14.38	4.89	15.76	13.1	8.14
IFI6	16.75	19.08	16.74	8.77	11.98	15.48	10.5	17.23	10.18
IRF5	16.75	6.73	5.86	3.51	9.59	5.7	8.75	5.51	6.11
MFN2	16.75	16.84	22.59	12.28	25.17	26.88	29.76	30.33	23.41
MTHFD2	16.75	14.59	22.59	14.03	37.15	18.74	12.26	18.61	14.25
PDHA1	16.75	15.71	20.08	19.29	22.77	17.92	10.5	17.92	18.32
PPP2R5D	16.75	7.86	15.06	14.03	13.18	15.48	17.51	25.5	19.34
SLC25A20	16.75	11.22	7.53	8.77	4.79	11.4	14.01	34.47	20.35
TICAM1	16.75	37.04	20.08	5.26	22.77	13.85	15.76	15.16	10.18
TRAF3	16.75	13.47	21.76	19.29	31.16	17.92	19.26	17.92	10.18
TRAV38-1	16.75	5.61	14.22	17.54	5.99	15.48	14.01	8.27	9.16
CBR4	15.92	13.47	15.06	10.52	10.79	9.77	12.26	13.1	18.32
CCR2	15.92	4.49	8.37	7.01	2.4	1	8.75	14.48	5.09
CD28	15.92	15.71	21.76	24.55	27.56	26.07	22.76	17.92	23.41
HDAC8	15.92	11.22	9.2	15.78	14.38	13.03	5.25	15.16	8.14
IL1RN	15.92	39.28	5.86	8.77	10.79	6.52	12.26	7.58	11.19
IRF4	15.92	21.33	15.06	17.54	41.94	16.29	14.01	25.5	38.67
MAP3K14	15.92	15.71	18.41	17.54	10.79	18.74	7	15.16	17.3
MTOR	15.92	19.08	19.25	19.29	22.77	22.81	28.01	24.13	19.34
OMA1	15.92	13.47	16.74	17.54	7.19	12.22	19.26	15.85	20.35
PDK3	15.92	11.22	18.41	21.04	14.38	19.55	24.51	19.99	17.3
PRKAB2	15.92	12.35	25.94	15.78	10.79	14.66	21.01	22.75	18.32
STAM	15.92	10.1	13.39	10.52	14.38	20.36	15.76	14.48	15.27
TFB2M	15.92	20.2	16.74	7.01	4.79	17.11	29.76	8.96	12.21
TRAV3	15.92	5.61	15.9	12.28	8.39	9.77	15.76	16.54	8.14
TRBV15	15.92	7.86	7.53	19.29	8.39	13.03	14.01	11.03	13.23
TRBV7-3	15.92	7.86	24.27	19.29	16.78	13.03	17.51	8.96	19.34
XCL1/2	15.92	4.49	9.2	17.54	34.75	30.14	21.01	20.68	20.35
ARAF	15.08	23.57	15.9	12.28	17.98	12.22	28.01	14.48	9.16
CASP3	15.08	12.35	15.9	12.28	19.17	19.55	28.01	18.61	29.51

CISH	15.08	11.22	10.04	8.77	4.79	3.26	10.5	11.03	7.12
MAML3	15.08	10.1	13.39	8.77	1.2	6.52	10.5	4.14	5.09
MS4A1	15.08	12.35	22.59	29.81	11.98	29.32	36.77	8.27	6.11
NSD2	15.08	17.96	23.43	26.31	20.37	25.25	26.26	36.53	23.41
SLC7A5	15.08	72.96	17.57	21.04	192.94	39.91	45.52	13.79	25.44
TOMM6	15.08	16.84	10.88	17.54	19.17	10.59	21.01	11.03	12.21
TOX	15.08	12.35	15.9	14.03	10.79	18.74	19.26	19.99	27.48
TRBV6-6	15.08	5.61	10.04	14.03	9.59	3.26	14.01	28.26	12.21
CCR4	14.24	8.98	5.02	8.77	38.35	8.96	12.26	19.99	17.3
CKAP5	14.24	10.1	12.55	5.26	11.98	7.33	3.5	13.1	10.18
FCAR	14.24	7.86	7.53	8.77	4.79	12.22	10.5	4.14	7.12
PIK3R2	14.24	7.86	13.39	12.28	9.59	5.7	3.5	6.2	9.16
PTGS2	14.24	44.9	2.51	7.01	5.99	6.52	22.76	7.58	8.14
TFDP1	14.24	15.71	14.22	17.54	11.98	26.07	26.26	32.4	30.53
TRBV10-3	14.24	11.22	22.59	21.04	9.59	11.4	38.52	26.19	6.11
TRBV13	14.24	3.37	10.88	14.03	11.98	13.85	12.26	19.99	3.05
TRBV2	14.24	11.22	17.57	10.52	21.57	19.55	14.01	22.75	18.32
TRBV6-5	14.24	12.35	26.78	29.81	10.79	25.25	22.76	35.16	18.32
TRBV7-8	14.24	15.71	15.06	50.86	13.18	24.44	19.26	15.16	30.53
STK11IP	14.24	16.84	10.88	8.77	14.38	8.96	5.25	6.2	13.23
ACVR1B	13.4	12.35	17.57	10.52	9.59	8.96	17.51	7.58	6.11
AREG	13.4	26.94	16.74	12.28	38.35	39.91	26.26	8.27	11.19
BCL2L1	13.4	13.47	14.22	8.77	5.99	10.59	14.01	20.68	18.32
CD163	13.4	4.49	2.51	1.75	3.6	4.07	1.75	3.45	3.05
CD1D	13.4	3.37	3.35	3.51	3.6	6.52	8.75	3.45	9.16
CD300LF	13.4	5.61	17.57	3.51	1.2	8.15	1.75	2.76	3.05
HMGCR	13.4	6.73	10.04	10.52	15.58	12.22	15.76	6.89	11.19
ICOS	13.4	16.84	19.25	22.8	65.91	21.99	15.76	25.5	29.51
IGF1R	13.4	13.47	22.59	26.31	11.98	18.74	14.01	10.34	7.12
PPAT	13.4	8.98	20.08	10.52	14.38	7.33	7	11.72	10.18
RARG	13.4	12.35	10.88	12.28	13.18	12.22	17.51	11.72	14.25
TNFRSF10B	13.4	10.1	18.41	14.03	23.97	13.85	15.76	7.58	12.21
TRAV12-1	13.4	10.1	17.57	22.8	13.18	15.48	12.26	24.13	20.35
TRAV24	13.4	52.75	7.53	7.01	4.79	7.33	10.5	8.27	5.09
TRAV38-2	13.4	5.61	14.22	21.04	7.19	4.89	14.01	3.45	7.12
CD160	12.56	6.73	10.04	5.26	3.6	8.15	8.75	13.79	11.19
CD33	12.56	3.37	7.53	1.75	7.19	1	8.75	4.83	1.02
GNG10	12.56	8.98	6.69	5.26	4.79	8.15	8.75	8.27	9.16
HDAC7	12.56	7.86	20.08	26.31	9.59	11.4	12.26	8.96	10.18
HK2	12.56	8.98	9.2	3.51	3.6	5.7	10.5	5.51	7.12
HK3	12.56	6.73	11.71	3.51	1.2	8.96	5.25	6.2	5.09
IL18BP	12.56	10.1	15.06	5.26	13.18	15.48	7	13.79	16.28
PPARA	12.56	8.98	11.71	5.26	7.19	10.59	14.01	6.2	10.18
PPP3CA	12.56	11.22	15.9	12.28	20.37	18.74	14.01	15.16	15.27

RNASEL	12.56	8.98	8.37	8.77	5.99	4.89	7	2.76	9.16
SMAD5	12.56	6.73	20.08	12.28	14.38	10.59	12.26	8.27	15.27
SOCS5	12.56	8.98	15.06	19.29	11.98	17.92	8.75	6.89	10.18
TRAV13-1	12.56	5.61	33.47	29.81	11.98	17.92	26.26	22.75	28.5
TRAV23	12.56	8.98	23.43	10.52	4.79	7.33	10.5	8.27	5.09
TRAV4	12.56	16.84	20.08	17.54	17.98	13.03	10.5	24.82	15.27
TRAV8-6	12.56	6.73	12.55	15.78	5.99	16.29	21.01	8.27	5.09
TRBV12-3	12.56	10.1	10.04	17.54	8.39	14.66	14.01	12.41	9.16
TRBV5-1	12.56	10.1	6.69	12.28	7.19	9.77	3.5	6.89	7.12
TRBV6-2	12.56	6.73	21.76	12.28	10.79	11.4	12.26	26.19	17.3
CD38	11.73	8.98	5.86	5.26	5.99	7.33	10.5	31.02	10.18
CMKLR1	11.73	4.49	5.02	5.26	2.4	4.89	1.75	6.89	19.34
FAS	11.73	17.96	15.06	10.52	17.98	16.29	10.5	21.37	25.44
IL18	11.73	12.35	6.69	12.28	2.4	4.07	7	1.38	6.11
IRF8	11.73	11.22	7.53	3.51	9.59	9.77	12.26	2.76	8.14
MTHFR	11.73	7.86	10.04	15.78	9.59	6.52	15.76	9.65	9.16
NCAPG2	11.73	16.84	9.2	5.26	8.39	5.7	12.26	13.1	14.25
NCR1	11.73	5.61	6.69	1.75	8.39	10.59	3.5	15.16	5.09
SMC2	11.73	11.22	9.2	5.26	5.99	8.96	21.01	19.3	13.23
SOCS2	11.73	13.47	12.55	8.77	13.18	11.4	14.01	15.85	12.21
TAOK2	11.73	6.73	11.71	7.01	9.59	10.59	7	12.41	14.25
TRAV26-2	11.73	8.98	9.2	1.75	5.99	5.7	5.25	4.14	11.19
TRAV5	11.73	7.86	12.55	7.01	4.79	6.52	7	13.1	5.09
TRBV4-2	11.73	10.1	14.22	12.28	14.38	8.15	10.5	22.06	8.14
TRBV6-1	11.73	7.86	5.02	7.01	7.19	6.52	3.5	15.16	7.12
ZBTB16	11.73	22.45	5.02	7.01	9.59	9.77	12.26	29.64	12.21
TBC1D10B	11.73	22.45	5.86	14.03	5.99	8.96	17.51	15.85	7.12
APC	10.89	7.86	11.71	15.78	3.6	8.96	19.26	14.48	7.12
CD9	10.89	7.86	12.55	15.78	14.38	6.52	21.01	7.58	9.16
CIITA	10.89	3.37	6.69	8.77	5.99	5.7	1.75	4.14	4.07
CMIP	10.89	7.86	5.86	7.01	10.79	12.22	5.25	6.2	5.09
CTLA4	10.89	6.73	13.39	8.77	19.17	8.15	14.01	33.78	43.76
IDH3A	10.89	20.2	12.55	12.28	10.79	13.85	26.26	19.3	19.34
IL6R	10.89	3.37	5.86	7.01	8.39	5.7	1.75	2.07	4.07
NFAT5	10.89	17.96	7.53	14.03	7.19	13.85	7	17.92	17.3
OAS3	10.89	16.84	15.06	7.01	5.99	8.15	10.5	12.41	8.14
PCCA	10.89	10.1	8.37	8.77	2.4	8.15	3.5	5.51	9.16
SGK3	10.89	12.35	17.57	12.28	13.18	16.29	19.26	7.58	10.18
TLR4	10.89	13.47	5.86	7.01	5.99	5.7	3.5	2.76	3.05
TRAV2	10.89	8.98	14.22	31.57	14.38	19.55	19.26	21.37	12.21
TRAV8-1	10.89	10.1	12.55	14.03	11.98	9.77	19.26	17.92	7.12
TRDV1	10.89	7.86	10.88	15.78	7.19	10.59	15.76	9.65	9.16
VAV3	10.89	16.84	15.9	10.52	20.37	31.77	22.76	15.16	10.18
CD40LG	10.05	7.86	5.02	3.51	14.38	6.52	10.5	9.65	3.05

COX19	10.05	16.84	15.06	14.03	7.19	13.85	12.26	9.65	12.21
ENTPD1	10.05	6.73	5.86	1.75	5.99	4.07	10.5	6.2	6.11
HAVCR2	10.05	17.96	6.69	3.51	7.19	17.11	8.75	15.85	7.12
IFI35	10.05	13.47	10.04	12.28	5.99	5.7	15.76	6.89	10.18
IL23A	10.05	11.22	12.55	12.28	15.58	4.89	12.26	6.89	6.11
LTA	10.05	11.22	15.06	7.01	26.36	5.7	24.51	13.79	8.14
PRICKLE3	10.05	11.22	8.37	3.51	13.18	9.77	7	7.58	2.04
TNFSF9	10.05	16.84	13.39	14.03	39.55	15.48	15.76	8.27	21.37
TRAV16	10.05	5.61	15.9	21.04	8.39	8.15	7	9.65	10.18
TRAV20	10.05	13.47	10.04	17.54	7.19	10.59	15.76	11.72	9.16
TRAV21	10.05	1.12	11.71	5.26	1.2	10.59	8.75	6.89	11.19
TRBV11-2	10.05	15.71	12.55	19.29	19.17	10.59	28.01	27.57	11.19
TRBV18	10.05	2.24	4.18	1.75	2.4	10.59	12.26	2.76	2.04
TRBV24-1	10.05	10.1	6.69	8.77	2.4	2.44	5.25	2.76	4.07
TRGV4	10.05	5.61	4.18	3.51	5.99	10.59	1.75	6.2	6.11
ACAD10	9.21	15.71	11.71	7.01	13.18	7.33	12.26	8.96	8.14
BTBD6	9.21	10.1	15.06	8.77	13.18	9.77	10.5	11.72	11.19
CCL25	9.21	7.86	9.2	8.77	11.98	6.52	10.5	10.34	4.07
CD300C	9.21	11.22	10.04	1.75	2.4	4.89	3.5	3.45	3.05
CPT1A	9.21	5.61	5.86	8.77	3.6	11.4	12.26	19.99	14.25
IL13	9.21	4.49	1.67	1.75	1.2	4.89	7	5.51	2.04
IL7	9.21	8.98	8.37	8.77	7.19	4.89	14.01	4.14	6.11
KLRD1	9.21	37.04	9.2	15.78	3.6	17.11	21.01	39.98	30.53
SRR	9.21	4.49	8.37	5.26	3.6	5.7	1.75	6.2	6.11
TCL1A	9.21	4.49	5.86	3.51	2.4	5.7	1.75	2.76	5.09
TRAV9-2	9.21	15.71	13.39	22.8	16.78	11.4	12.26	13.1	16.28
TRBV6-4	9.21	3.37	2.51	12.28	11.98	2.44	12.26	8.96	3.05
ATP5MF	8.38	11.22	5.02	1.75	1.2	7.33	7	7.58	12.21
CLDN7	8.38	1.12	2.51	12.28	5.99	4.07	7	1	1.02
COLEC12	8.38	13.47	6.69	14.03	7.19	4.89	3.5	2.07	6.11
MMP9	8.38	10.1	4.18	12.28	10.79	5.7	14.01	5.51	6.11
NEK2	8.38	8.98	6.69	7.01	4.79	4.89	7	8.27	12.21
NOD2	8.38	6.73	5.02	1.75	3.6	5.7	3.5	2.07	6.11
RDH14	8.38	6.73	16.74	14.03	9.59	8.15	19.26	16.54	8.14
SELPLG	8.38	5.61	11.71	3.51	11.98	9.77	10.5	5.51	13.23
SHMT1	8.38	12.35	6.69	10.52	8.39	5.7	8.75	6.89	5.09
SHMT2	8.38	11.22	7.53	17.54	10.79	6.52	14.01	15.16	10.18
TNFRSF4	8.38	6.73	3.35	7.01	16.78	3.26	14.01	4.14	1.02
TRAV25	8.38	3.37	7.53	10.52	7.19	3.26	5.25	9.65	6.11
TRAV26-1	8.38	8.98	16.74	10.52	7.19	16.29	14.01	11.72	7.12
TRAV29	8.38	6.73	15.06	8.77	9.59	13.03	26.26	4.14	15.27
TRBV7-2	8.38	10.1	37.65	15.78	21.57	8.15	21.01	41.36	25.44
TRDV2	8.38	3.37	1.67	1.75	1.2	1	1.75	31.02	10.18
TRGV9	8.38	2.24	2.51	5.26	2.4	3.26	1.75	2.07	3.05

BATF	7.54	4.49	3.35	3.51	20.37	5.7	10.5	9.65	7.12
BUB1	7.54	7.86	10.88	3.51	10.79	4.89	10.5	19.99	11.19
C3	7.54	7.86	5.86	5.26	5.99	4.89	8.75	6.2	2.04
CLCF1	7.54	15.71	9.2	14.03	5.99	8.96	12.26	10.34	3.05
CPT1B	7.54	11.22	22.59	10.52	5.99	9.77	3.5	13.1	15.27
CXCL1	7.54	4.49	2.51	3.51	2.4	1.63	3.5	4.83	2.04
CXCL3	7.54	24.69	8.37	7.01	10.79	11.4	8.75	2.76	10.18
FPR1	7.54	8.98	3.35	10.52	2.4	4.07	3.5	2.76	4.07
GOT1	7.54	8.98	5.86	5.26	7.19	5.7	3.5	6.2	5.09
ICOSLG	7.54	8.98	6.69	5.26	7.19	12.22	7	6.89	6.11
KLRF1	7.54	4.49	6.69	5.26	5.99	4.07	5.25	26.19	3.05
LAG3	7.54	14.59	12.55	8.77	13.18	12.22	10.5	35.16	25.44
MKI67	7.54	11.22	10.04	15.78	8.39	9.77	12.26	39.29	28.5
PHGDH	7.54	13.47	5.02	12.28	10.79	8.15	21.01	6.89	13.23
PLCB3	7.54	7.86	5.02	5.26	3.6	5.7	7	2.07	4.07
PTGS1	7.54	8.98	9.2	5.26	3.6	4.07	3.5	3.45	6.11
RORC	7.54	2.24	4.18	10.52	1.2	3.26	5.25	16.54	4.07
RPTOR	7.54	5.61	10.04	5.26	7.19	10.59	8.75	8.27	4.07
TRAV13-2	7.54	10.1	12.55	7.01	7.19	8.96	8.75	13.79	13.23
TRBV14	7.54	22.45	10.04	14.03	10.79	17.11	10.5	12.41	11.19
TRBV3-1	7.54	7.86	10.88	8.77	8.39	12.22	12.26	13.79	5.09
WDR45	7.54	3.37	12.55	17.54	16.78	9.77	12.26	7.58	9.16
CD19	6.7	5.61	3.35	7.01	4.79	4.89	5.25	4.83	2.04
COX16	6.7	6.73	8.37	12.28	7.19	5.7	8.75	7.58	9.16
DHFR2	6.7	5.61	4.18	1.75	1.2	4.89	3.5	3.45	2.04
GFER	6.7	8.98	7.53	3.51	9.59	6.52	8.75	8.96	12.21
GRPEL1	6.7	16.84	20.08	7.01	5.99	10.59	12.26	4.83	8.14
IL23R	6.7	10.1	10.04	7.01	9.59	3.26	7	8.27	5.09
KIR3DL1/2	6.7	25.82	7.53	33.32	9.59	39.91	21.01	6.89	10.18
LILRB4	6.7	5.61	6.69	1.75	5.99	1	8.75	2.76	1.02
MAPK12	6.7	7.86	3.35	7.01	5.99	1.63	3.5	2.07	1.02
MCAT	6.7	8.98	4.18	5.26	3.6	7.33	3.5	4.83	1.02
MMP2	6.7	4.49	3.35	8.77	7.19	4.07	3.5	5.51	5.09
MR1	6.7	8.98	5.02	7.01	4.79	9.77	8.75	11.72	7.12
MYL9	6.7	7.86	3.35	5.26	3.6	4.07	3.5	1.38	9.16
NBL1	6.7	17.96	3.35	8.77	26.36	3.26	8.75	3.45	5.09
NDUFA2	6.7	6.73	8.37	7.01	2.4	9.77	10.5	8.27	8.14
NRF1	6.7	13.47	5.86	12.28	13.18	6.52	14.01	18.61	8.14
PIDD1	6.7	13.47	11.71	8.77	14.38	13.85	10.5	7.58	8.14
RDH10	6.7	6.73	5.86	5.26	7.19	6.52	14.01	6.89	7.12
SIGLEC5	6.7	12.35	6.69	17.54	9.59	4.89	3.5	4.14	4.07
SLC2A1	6.7	12.35	9.2	3.51	9.59	7.33	8.75	2.76	6.11
TNFSF13B	6.7	7.86	4.18	5.26	4.79	11.4	10.5	4.14	6.11
TRAV27	6.7	4.49	8.37	8.77	2.4	5.7	5.25	10.34	10.18

TRAV35	6.7	2.24	12.55	14.03	2.4	1.63	8.75	5.51	7.12
TRIM10	6.7	5.61	5.86	3.51	7.19	3.26	7	6.89	5.09
ACSF2	5.86	3.37	5.86	3.51	5.99	1	7	2.07	2.04
B3GAT1	5.86	8.98	3.35	5.26	1.2	1.63	3.5	1.38	4.07
BATF3	5.86	6.73	7.53	5.26	3.6	8.96	10.5	1.38	3.05
CCL2	5.86	5.61	4.18	1.75	3.6	1	1.75	2.76	4.07
CD1A	5.86	3.37	3.35	1.75	3.6	1.63	3.5	2.76	4.07
CLDN23	5.86	4.49	5.02	5.26	5.99	3.26	12.26	1	4.07
CRLF2	5.86	10.1	6.69	5.26	3.6	5.7	8.75	4.14	8.14
CX3CL1	5.86	8.98	5.86	3.51	7.19	2.44	7	4.83	3.05
CXCL5	5.86	6.73	3.35	3.51	5.99	4.89	3.5	1.38	1.02
FASLG	5.86	4.49	2.51	5.26	7.19	4.07	3.5	6.89	9.16
IL15	5.86	5.61	10.04	12.28	2.4	9.77	8.75	4.83	6.11
LEFTY1	5.86	2.24	2.51	3.51	3.6	3.26	1.75	2.07	5.09
MS4A4A	5.86	6.73	14.22	8.77	1.2	4.89	3.5	2.76	4.07
NFIL3	5.86	17.96	4.18	5.26	2.4	8.96	19.26	4.83	5.09
PPARD	5.86	3.37	1	5.26	4.79	8.15	7	8.27	1.02
RAG2	5.86	12.35	2.51	7.01	9.59	6.52	17.51	4.83	5.09
SRC	5.86	11.22	7.53	3.51	1.2	4.07	3.5	2.76	3.05
TRAV10	5.86	5.61	14.22	10.52	8.39	18.74	10.5	17.92	3.05
TRAV6	5.86	2.24	8.37	8.77	4.79	4.07	8.75	6.89	5.09
TRBV30	5.86	11.22	10.88	10.52	17.98	12.22	5.25	8.96	1.02
ACACA	5.03	2.24	7.53	5.26	7.19	4.89	8.75	6.2	2.04
ACOT2	5.03	15.71	6.69	21.04	10.79	12.22	14.01	12.41	9.16
ACVR1C	5.03	4.49	3.35	1.75	4.79	7.33	5.25	4.14	4.07
AFDN	5.03	3.37	1.67	10.52	5.99	8.96	8.75	2.07	5.09
CCL11	5.03	2.24	5.86	1.75	4.79	4.07	7	1.38	3.05
CCL24	5.03	8.98	6.69	7.01	9.59	1	10.5	3.45	6.11
CCL28	5.03	11.22	10.04	5.26	8.39	16.29	21.01	4.83	9.16
CD244	5.03	10.1	8.37	1.75	2.4	3.26	3.5	6.2	5.09
CD274	5.03	8.98	4.18	1.75	3.6	4.89	12.26	6.89	2.04
CLDN4	5.03	12.35	10.04	14.03	8.39	8.15	12.26	11.03	5.09
CXCR5	5.03	11.22	5.02	8.77	11.98	8.96	26.26	6.89	4.07
EOMES	5.03	2.24	3.35	8.77	4.79	2.44	3.5	8.96	5.09
GLS2	5.03	4.49	5.86	3.51	7.19	6.52	7	2.07	3.05
IFIT2	5.03	8.98	5.02	14.03	4.79	7.33	10.5	4.83	5.09
IKZF4	5.03	3.37	5.86	3.51	3.6	4.07	5.25	4.83	1.02
IL12RB2	5.03	3.37	5.02	8.77	3.6	5.7	5.25	8.27	2.04
IRF6	5.03	5.61	1.67	5.26	3.6	8.96	7	1.38	2.04
KYAT1	5.03	15.71	11.71	7.01	5.99	3.26	7	5.51	4.07
NCAPH	5.03	14.59	3.35	8.77	4.79	9.77	17.51	15.85	19.34
PFKFB4	5.03	10.1	5.86	8.77	1.2	2.44	10.5	2.07	2.04
PIK3R3	5.03	2.24	2.51	5.26	7.19	1.63	7	5.51	7.12
PTGDR2	5.03	4.49	3.35	1.75	1.2	2.44	3.5	2.76	1.02

RAI1	5.03	7.86	11.71	7.01	5.99	8.15	5.25	8.27	5.09
SLC2A11	5.03	10.1	15.06	7.01	2.4	8.15	7	5.51	6.11
SPIB	5.03	6.73	2.51	5.26	2.4	6.52	3.5	2.76	3.05
TRAV30	5.03	7.86	3.35	10.52	3.6	5.7	1.75	1	7.12
CCL18	4.19	4.49	4.18	7.01	2.4	4.89	1.75	1	1.02
CCL7	4.19	7.86	4.18	1.75	3.6	3.26	7	2.76	1.02
CCR5	4.19	3.37	5.02	1.75	1.2	1.63	10.5	28.95	18.32
CD276	4.19	7.86	6.69	12.28	4.79	4.07	8.75	4.14	4.07
CD40	4.19	6.73	4.18	7.01	3.6	4.07	5.25	2.07	3.05
CLDN1	4.19	3.37	3.35	1.75	4.79	8.15	8.75	2.07	9.16
CXCL16	4.19	28.06	23.43	12.28	9.59	16.29	12.26	2.76	5.09
ENTPD2	4.19	5.61	2.51	8.77	2.4	4.07	5.25	2.76	10.18
IL10	4.19	2.24	4.18	3.51	2.4	1.63	10.5	4.83	4.07
IL2	4.19	8.98	1	7.01	5.99	3.26	10.5	3.45	3.05
IL21	4.19	2.24	3.35	5.26	3.6	1	1.75	1	3.05
IL27	4.19	3.37	3.35	8.77	2.4	1.63	1.75	2.07	3.05
IL2RA	4.19	4.49	1.67	5.26	3.6	4.07	7	1.38	1.02
IL33	4.19	6.73	5.02	3.51	3.6	2.44	17.51	4.14	4.07
IL36A	4.19	1.12	2.51	1.75	1.2	4.89	5.25	1	3.05
IL36B	4.19	4.49	4.18	1.75	4.79	4.89	1.75	4.14	6.11
IL3RA	4.19	3.37	3.35	1.75	5.99	1	3.5	1	1.02
IL6	4.19	40.41	5.02	5.26	9.59	3.26	15.76	2.07	3.05
IL9	4.19	4.49	2.51	1.75	2.4	2.44	3.5	2.07	2.04
MTHFD1L	4.19	5.61	10.04	14.03	11.98	16.29	17.51	11.72	7.12
NCAM1	4.19	16.84	5.86	1.75	3.6	4.89	12.26	8.27	5.09
OXSM	4.19	7.86	9.2	7.01	4.79	10.59	7	5.51	2.04
PTCD1	4.19	7.86	6.69	3.51	1.2	4.89	1.75	6.2	5.09
PYCR1	4.19	6.73	5.02	8.77	7.19	4.89	5.25	4.14	2.04
RDH5	4.19	2.24	4.18	7.01	5.99	4.89	10.5	2.07	1.02
TLR9	4.19	2.24	8.37	3.51	3.6	4.89	3.5	2.07	4.07
TNFRSF11A	4.19	1.12	1.67	3.51	2.4	1.63	1.75	1	2.04
TPSAB1/B2	4.19	7.86	2.51	5.26	2.4	4.07	8.75	4.83	7.12
TRAV11	4.19	5.61	3.35	1.75	8.39	3.26	7	2.76	1.02
TRAV22	4.19	7.86	7.53	5.26	1.2	2.44	12.26	13.1	7.12
TRAV39	4.19	5.61	10.88	5.26	2.4	4.89	3.5	2.07	3.05
TRBV11-1	4.19	4.49	4.18	3.51	8.39	4.07	1.75	1	2.04
TRBV5-6	4.19	6.73	4.18	5.26	9.59	9.77	10.5	5.51	6.11
TRBV7-6	4.19	11.22	9.2	17.54	11.98	8.96	1.75	6.89	18.32
ANGPT2	3.35	2.24	1.67	7.01	1.2	4.89	3.5	2.07	1.02
BLK	3.35	6.73	1.67	7.01	3.6	8.15	8.75	4.83	9.16
CARD10	3.35	3.37	3.35	7.01	2.4	2.44	3.5	2.76	5.09
CCL1	3.35	13.47	3.35	1.75	8.39	5.7	3.5	4.83	4.07
CCL13	3.35	3.37	3.35	1.75	1.2	1	1.75	2.07	1.02
CCL14	3.35	2.24	2.51	3.51	2.4	2.44	8.75	2.07	6.11

CCL8	3.35	5.61	5.02	5.26	7.19	4.07	1.75	2.07	2.04
CD200	3.35	6.73	2.51	3.51	3.6	4.07	8.75	5.51	7.12
CD22	3.35	2.24	1	22.8	1.2	4.07	10.5	2.07	2.04
CD80	3.35	2.24	3.35	5.26	4.79	3.26	5.25	1	7.12
CLDN3	3.35	1.12	4.18	10.52	3.6	2.44	1.75	1	5.09
CXCL14	3.35	2.24	1	8.77	2.4	2.44	8.75	3.45	1.02
CXCR6	3.35	2.24	6.69	5.26	9.59	4.07	14.01	22.06	5.09
EGF	3.35	4.49	3.35	3.51	4.79	3.26	5.25	3.45	3.05
FAM30A	3.35	2.24	4.18	5.26	1.2	2.44	7	1	4.07
FKBP1A	3.35	6.73	6.69	7.01	1.2	6.52	5.25	6.2	5.09
IFNA1	3.35	3.37	1	1.75	1.2	1	5.25	3.45	1.02
IL12A	3.35	7.86	5.86	5.26	4.79	4.89	5.25	6.89	5.09
IL1A	3.35	8.98	1.67	5.26	4.79	1.63	3.5	2.07	7.12
IL31	3.35	7.86	2.51	3.51	4.79	1.63	5.25	1	2.04
IL36RN	3.35	1.12	1.67	3.51	1.2	2.44	3.5	1	1.02
IL37	3.35	1.12	1	3.51	3.6	1.63	5.25	1	1.02
MTCP1	3.35	6.73	5.02	3.51	8.39	2.44	1.75	2.07	5.09
PNOC	3.35	5.61	1	7.01	3.6	2.44	1.75	2.76	1.02
PPT2	3.35	2.24	5.02	12.28	4.79	7.33	1.75	2.76	10.18
PSAT1	3.35	3.37	5.86	10.52	2.4	4.07	3.5	6.89	7.12
PSPH	3.35	4.49	4.18	1.75	3.6	4.07	7	1	5.09
SGO2	3.35	3.37	4.18	3.51	3.6	4.89	3.5	1.38	3.05
TNFRSF17	3.35	4.49	1	1.75	1.2	1	3.5	1.38	2.04
TRAV36	3.35	6.73	1.67	5.26	1.2	4.07	3.5	5.51	7.12
TRAV7	3.35	7.86	2.51	1.75	3.6	4.89	1.75	2.07	1.02
TRAV9-1	3.35	12.35	3.35	5.26	2.4	2.44	1.75	5.51	3.05
TRBV12-5	3.35	3.37	4.18	3.51	3.6	4.89	3.5	4.83	7.12
TRBV5-4	3.35	1.12	5.02	5.26	2.4	1	3.5	4.14	2.04
TRBV6-9	3.35	6.73	1.67	12.28	8.39	5.7	5.25	2.07	1.02
TRIM31	3.35	2.24	4.18	3.51	2.4	1	5.25	4.83	6.11
TRIM34	3.35	2.24	4.18	1.75	8.39	6.52	5.25	6.2	1.02
ALDH1L1	2.51	2.24	4.18	5.26	1.2	3.26	1.75	1.38	1.02
ALDH8A1	2.51	4.49	3.35	3.51	5.99	8.15	7	6.2	6.11
AURKA	2.51	7.86	7.53	7.01	7.19	1.63	3.5	5.51	6.11
CCL15	2.51	6.73	4.18	3.51	1.2	4.07	1.75	2.76	2.04
CCL19	2.51	2.24	1	5.26	2.4	4.89	3.5	3.45	3.05
CCL22	2.51	3.37	5.86	3.51	1.2	1.63	5.25	5.51	4.07
CCL23	2.51	1.12	2.51	3.51	2.4	1	7	1	1.02
CCL26	2.51	2.24	1.67	1.75	5.99	1.63	3.5	1	1.02
CCL27	2.51	3.37	2.51	3.51	4.79	2.44	3.5	1	1.02
CCR6	2.51	7.86	2.51	7.01	8.39	5.7	5.25	7.58	6.11
CCR8	2.51	4.49	2.51	1.75	2.4	1	1.75	1.38	1.02
CD86	2.51	6.73	5.02	1.75	3.6	4.07	3.5	1.38	3.05
CEACAM3	2.51	3.37	1	1.75	1.2	2.44	1.75	1.38	1.02

CSF3	2.51	7.86	4.18	1.75	2.4	3.26	1.75	4.14	3.05
CXCL10	2.51	1.12	5.86	3.51	2.4	2.44	1.75	3.45	2.04
DLL1	2.51	2.24	1	7.01	8.39	8.96	3.5	5.51	1.02
FCRL2	2.51	3.37	1	1.75	3.6	4.07	15.76	1	2.04
FOXP3	2.51	2.24	5.86	1.75	8.39	2.44	10.5	6.89	1.02
GRK4	2.51	5.61	1.67	3.51	3.6	1	8.75	4.83	3.05
HDC	2.51	3.37	6.69	3.51	4.79	4.07	8.75	4.14	2.04
IFIT3	2.51	13.47	1	5.26	1.2	1.63	5.25	1.38	3.05
IL17A	2.51	1.12	2.51	3.51	1.2	3.26	3.5	1	2.04
IL1F10	2.51	3.37	1.67	1.75	2.4	1.63	7	1	1.02
IL22	2.51	1.12	2.51	1.75	2.4	1.63	7	2.07	4.07
LNX1	2.51	1.12	5.86	1.75	1.2	1.63	1.75	1	1.02
MS4A2	2.51	2.24	1	1.75	2.4	1.63	1.75	3.45	1.02
NRL	2.51	5.61	6.69	7.01	2.4	4.07	7	3.45	7.12
OASL	2.51	19.08	1.67	1.75	10.79	7.33	5.25	12.41	18.32
PDCD1	2.51	1.12	4.18	5.26	7.19	8.15	3.5	4.14	2.04
PPARGC1A	2.51	3.37	4.18	1.75	3.6	4.07	5.25	2.07	1.02
TERT	2.51	5.61	3.35	1.75	4.79	2.44	3.5	2.07	1.02
TNFRSF18	2.51	3.37	2.51	7.01	4.79	3.26	3.5	4.14	7.12
TNFRSF9	2.51	2.24	3.35	7.01	7.19	2.44	3.5	1.38	7.12
TRAV18	2.51	1.12	1.67	1.75	1.2	3.26	1.75	2.07	2.04
TRAV40	2.51	2.24	1.67	5.26	2.4	1.63	1.75	1.38	1.02
TRBV11-3	2.51	2.24	1	1.75	1.2	1.63	1.75	1.38	1.02
TRBV25-1	2.51	5.61	6.69	10.52	2.4	4.89	15.76	8.96	8.14
TRBV5-5	2.51	7.86	4.18	1.75	5.99	2.44	1.75	4.14	6.11
TRBV7-4	2.51	4.49	10.04	21.04	3.6	3.26	7	3.45	3.05
TRBV7-7	2.51	2.24	1	5.26	1.2	4.89	5.25	4.14	2.04
USP18	2.51	5.61	4.18	1.75	3.6	1.63	1.75	1.38	6.11
CCL17	1.68	1.12	2.51	1.75	2.4	1	3.5	2.07	2.04
CPA3	1.68	2.24	1.67	1.75	1.2	1	1.75	1.38	1.02
CXCL12	1.68	4.49	1.67	7.01	8.39	2.44	12.26	2.07	3.05
CXCL9	1.68	7.86	1.67	1.75	2.4	1.63	1.75	1	2.04
EBI3	1.68	1.12	1	1.75	1.2	1.63	1.75	2.76	1.02
HSD11B1	1.68	6.73	2.51	3.51	4.79	1.63	1.75	3.45	2.04
IDO1	1.68	11.22	2.51	1.75	1.2	1.63	7	2.76	1.02
IDO2	1.68	1.12	2.51	1.75	1.2	1.63	3.5	2.76	1.02
IFIT1	1.68	1.12	4.18	3.51	1.2	1.63	5.25	1.38	1.02
IL11	1.68	1.12	1	1.75	4.79	1.63	1.75	1	1.02
IL17C	1.68	3.37	2.51	1.75	10.79	4.07	3.5	2.07	1.02
IL17F	1.68	3.37	2.51	5.26	1.2	2.44	5.25	1	1.02
IL19	1.68	1.12	1	5.26	1.2	1	1.75	2.76	1.02
IL1R1	1.68	2.24	1.67	1.75	3.6	2.44	3.5	1	3.05
IL26	1.68	1.12	4.18	7.01	3.6	3.26	1.75	2.76	1.02
IL3	1.68	4.49	7.53	1.75	3.6	4.07	1.75	3.45	5.09

IL5	1.68	4.49	2.51	1.75	3.6	1	1.75	1	3.05
JAG1	1.68	4.49	2.51	1.75	4.79	5.7	3.5	4.83	2.04
MINOS1	1.68	2.24	4.18	3.51	2.4	2.44	1.75	1	1.02
PDGFRA	1.68	1.12	2.51	3.51	4.79	1.63	5.25	1.38	3.05
PYCR3	1.68	3.37	3.35	1.75	1.2	1.63	7	1.38	1.02
SLC2A2	1.68	5.61	3.35	3.51	3.6	1	3.5	2.07	2.04
TRAV1-1	1.68	3.37	7.53	8.77	5.99	5.7	1.75	8.96	3.05
TRBV16	1.68	2.24	1	7.01	2.4	1.63	1.75	4.83	2.04
TRBV6-8	1.68	3.37	1.67	7.01	1.2	4.07	1.75	2.76	2.04
TRDV3	1.68	4.49	3.35	5.26	1.2	3.26	1.75	3.45	2.04
ACVR2A	1	2.24	4.18	1.75	2.4	6.52	5.25	2.07	5.09
ALDH1L2	1	5.61	1.67	1.75	2.4	3.26	1.75	2.76	1.02
CCL16	1	1.12	3.35	1.75	1.2	1	1.75	1	1.02
CCL21	1	1.12	2.51	1.75	1.2	1	1.75	1	1.02
CD209	1	1.12	4.18	1.75	2.4	1.63	5.25	2.07	1.02
CEACAM1	1	6.73	2.51	7.01	4.79	1	3.5	2.07	3.05
CSF2	1	5.61	1.67	1.75	1.2	1.63	1.75	1	1.02
CXCL11	1	2.24	1	3.51	2.4	1.63	1.75	1.38	1.02
CXCL13	1	3.37	2.51	1.75	2.4	1.63	1.75	2.07	1.02
CXCL6	1	3.37	1.67	1.75	2.4	1	1.75	1	1.02
IFNB1	1	3.37	1	1.75	1.2	1.63	1.75	1	1.02
IL12B	1	3.37	1.67	1.75	2.4	4.07	1.75	1	2.04
IL20	1	3.37	5.02	3.51	4.79	4.07	7	2.07	3.05
IL24	1	2.24	2.51	1.75	1.2	1.63	7	2.07	3.05
IL25	1	1.12	1	3.51	3.6	4.07	1.75	2.76	2.04
IL36G	1	1.12	1.67	1.75	2.4	1	3.5	2.07	1.02
IL4	1	4.49	1.67	1.75	2.4	1.63	1.75	1	1.02
LDHC	1	3.37	2.51	5.26	4.79	3.26	1.75	2.07	1.02
LIF	1	3.37	2.51	8.77	3.6	1	8.75	1	1.02
MDFIC	1	3.37	1.67	1.75	2.4	1.63	3.5	2.76	1.02
NCR2	1	6.73	2.51	5.26	2.4	1	3.5	2.07	5.09
PDCD1LG2	1	5.61	2.51	1.75	1.2	1.63	1.75	2.07	2.04
PDPN	1	1.12	2.51	1.75	1.2	2.44	3.5	2.76	1.02
PLA2G4B	1	4.49	1.67	1.75	1.2	1	1.75	1.38	1.02
PROCR	1	5.61	3.35	5.26	7.19	7.33	3.5	3.45	4.07
RARB	1	3.37	4.18	5.26	2.4	2.44	1.75	1	1.02
RIMKLA	1	3.37	1.67	1.75	2.4	1	1.75	1.38	1.02
SERINC4	1	6.73	5.02	3.51	3.6	4.07	12.26	1.38	4.07
TRAV34	1	3.37	2.51	1.75	1.2	1	1.75	2.76	1.02
TRBV10-1	1	2.24	1	1.75	1.2	1	3.5	1.38	3.05
TRBV10-2	1	8.98	7.53	15.78	2.4	11.4	12.26	7.58	5.09
TRBV5-8	1	2.24	1.67	1.75	7.19	3.26	3.5	3.45	3.05
